# Human microglial models to study HIV infection and neuropathogenesis: a literature overview and comparative analyses

**DOI:** 10.1007/s13365-021-01049-w

**Published:** 2022-02-09

**Authors:** Stephanie B. H. Gumbs, Raphael Kübler, Lavina Gharu, Pauline J. Schipper, Anne L. Borst, Gijsje J. L. J. Snijders, Paul R. Ormel, Amber Berdenis van Berlekom, Annemarie M. J. Wensing, Lot D. de Witte, Monique Nijhuis

**Affiliations:** 1 Translational Virology, Department of Medical Microbiology, University Medical Center Utrecht, Utrecht, The Netherlands; 2 Department of Psychiatry, Icahn School of Medicine, New York, NY USA; 3 Department of Psychiatry, University Medical Center Utrecht, Brain Center, Utrecht University, Utrecht, The Netherlands; 4 Department of Translational Neuroscience, University Medical Center Utrecht, Brain Center, Utrecht University, Utrecht, The Netherlands

**Keywords:** Microglia, HIV, HIV-associated neurocognitive disorder, Neuropathogenesis, Central nervous system, Organoid

## Abstract

HIV persistence in the CNS despite antiretroviral therapy may cause neurological disorders and poses a critical challenge for HIV cure. Understanding the pathobiology of HIV-infected microglia, the main viral CNS reservoir, is imperative. Here, we provide a comprehensive comparison of human microglial culture models: cultured primary microglia (pMG), microglial cell lines, monocyte-derived microglia (MDMi), stem cell–derived microglia (iPSC-MG), and microglia grown in 3D cerebral organoids (oMG) as potential model systems to advance HIV research on microglia. Functional characterization revealed phagocytic capabilities and responsiveness to LPS across all models. Microglial transcriptome profiles of uncultured pMG showed the highest similarity to cultured pMG and oMG, followed by iPSC-MG and then MDMi. Direct comparison of HIV infection showed a striking difference, with high levels of viral replication in cultured pMG and MDMi and relatively low levels in oMG resembling HIV infection observed in post-mortem biopsies, while the SV40 and HMC3 cell lines did not support HIV infection. Altogether, based on transcriptional similarities to uncultured pMG and susceptibility to HIV infection, MDMi may serve as a first screening tool, whereas oMG, cultured pMG, and iPSC-MG provide more representative microglial culture models for HIV research. The use of current human microglial cell lines (SV40, HMC3) is not recommended.

**Supplementary information:**

The online version contains supplementary material available at 10.1007/s13365-021-01049-w.

## Introduction

Currently, 38 million people are estimated to be living with HIV (www.who.int). Implementation of antiretroviral therapy (ART) resulted in effective suppression of viral replication and substantially reduced AIDS-related morbidity and mortality (Global HIV & AIDS Statistics 2020). However, ART neither eliminates HIV that persists in a latent state nor suppresses HIV expression and production from cellular reservoirs in the body (Sengupta and Siliciano 2018). Hence, despite long-term ART, HIV-1 persists in the central nervous system (CNS), which upon ART cessation contributes to the rekindling of viral infection and replication. Persistence of HIV in the CNS indirectly and directly results in a wide range of CNS manifestations in up to 50% of ART-treated HIV-infected individuals, collectively termed HIV-associated neurocognitive disorders (HAND) (Caruana et al. 2017; Heaton et al. 2010, 2011; Wang et al. 2020; Winston and Spudich 2020). In addition to viral factors, the onset or progression of HAND is exacerbated by systemic inflammation, myeloid activation, and a variety of common comorbid conditions including cardiovascular disease, chronic lung disease, diabetes, anemia, obesity, and substance abuse (Ances et al. 2021; Ances and Letendre 2019; Heaton et al. 2010, 2015). Besides optimizing the antiretroviral drug combination for better CNS penetration and encouraging the patients’ adherence to treatment, no other clear recommendation can be formulated for the treatment of HAND (Calcagno et al. 2017; Nosik et al. 2021; Ulfhammer et al. 2018; Winston and Spudich 2020). To develop novel therapeutic strategies that target this CNS reservoir and diminish HIV-associated pathogenesis in the CNS, it is therefore of primary interest to understand how HIV reservoirs in the brain are established and maintained.

HIV-1 enters the CNS within 2 to 4 weeks after initial infection. Viral RNA has been detected in the cerebrospinal fluid (CSF) as well as in brain tissues of both asymptomatic and symptomatic individuals (Davis et al. 1992; Enting et al. 2001; Pilcher et al. 2001; Tambussi et al. 2000). Later on, throughout the course of infection, compartmentalized HIV-1 populations, genetically distinct from viral populations replicating in the periphery, can be detected in the CSF or brain tissue of several HIV-infected individuals (Bednar et al. 2015; Harrington et al. 2009). Viral analyses showed that the CNS cells are predominantly infected by macrophage-tropic HIV-1 variants that utilize the CCR5 co-receptor and require a low density of the CD4 receptor for efficient cell entry and infection (R5 M-tropic) (Arrildt et al. 2015; Joseph et al. 2015; Joseph and Swanstrom 2018). Based on these cellular characteristics, microglia are thought to constitute the main viral reservoir in the CNS and support productive HIV infection allowing for viral compartmentalization, evolution and escape (Wallet et al. 2019).

Investigation of the pathobiology of HIV-infected microglia and how this potential viral reservoir is established and maintained is urgently needed but is restricted due to the difficulty of studying primary microglia. A great variety of microglial culture models have been developed in the past decade (Timmerman et al. 2018). The present study aimed to investigate the validity and similarity of these models to primary microglia in regard to HIV research. In the present study, we provide a comprehensive overview of five different human microglial culture models: cultured primary microglia (pMG), microglial cell lines (SV40, HMC3, C20), monocyte-derived microglia (MDMi), stem cell–derived microglia (iPSC-MG), and microglia grown in 3D cerebral organoids (oMG). For each model, we describe how it compares to human primary microglia in situ and in vitro across the characteristics morphology, gene expression, immune function, and HIV infection, as well as practical strengths and limitations. By using publicly available RNA-seq data, we evaluated the transcriptomic similarity of the models to uncultured primary microglia in the context of a microglia-specific core signature and HIV-relevant genes. Together, this study aims to provide researchers working on HIV with a guide to choosing a suitable microglial culture model for studying HIV infection and neuropathogenesis.

## Comprehensive overview: model description and comparison

### Description of the models

Isolation and culture of fetal and adult pMG from human brain tissue for in vitro studies has been performed already for a long time (Grenier et al. 1989; Hassan et al. 1991; Hayes et al. 1988; Lee et al. 1992). Microglia can be isolated by generating a cell suspension of the brain tissue followed by subsequent further microglia-specific enrichment techniques (Mizee et al. 2017; Olah et al. 2012; Rustenhoven et al. 2016; Zhang et al. 2016). Post isolation, cells can be cultured in vitro for weeks to months. Loss of phenotypic characteristics of primary microglia during isolation procedures is, however, well documented and known to aggravate once the cells are cultured (Gosselin et al. 2017; Marsh et al. 2020). Adding factors such as GM-CSF and IL-34 partly but not fully prevents this loss of phenotype (Gosselin et al. 2017). The limited availability of human brain tissue and the subsequent limited number of viable microglial cells present further difficulties in performing experiments with human pMG.

To combat these restrictions, pMG have been immortalized through viral transduction with different oncogenes to generate microglial human cell lines. Examples of these immortalized cell lines are HMC3, SV40, and C20 (Garcia-Mesa et al. 2017; Janabi et al. 1995). For over a decade, SV40 and HMC3 have been the only human microglial cell lines available. A comprehensive review of the HMC3 cell line was recently published by Dello Russo and colleagues reporting that the HMC3 cell line has been circulated and used in various laboratories with different denominations, i.e., C13NJ, CHME-3, and CHME-5 cells (Dello Russo et al. 2018). Notably, a recent study has reported that some of the circulating HMC3-based cell lines are impure, as the cells are of rat origin (Garcia-Mesa et al. 2017).

The natural plasticity of monocytes has been exploited in vitro to differentiate these cells towards dendritic cells and macrophages (Davies and Lloyd 1989; Zhou and Tedder 1996). These differentiated cells have been extensively used to study HIV biology (Ancuta et al. 2006; Ciborowski et al. 2007; Fairman and Angel 2012; Suzuki et al. 2000; Taya et al. 2014; Tsunetsugu-Yokota et al. 1995; Tuttle et al. 1998). Several protocols have been developed to direct monocytes towards a microglia-like phenotype, within 10 to 14 days, referred to as monocyte-derived microglia (MDMi). The generation of MDMi is based on the culture of monocytes in either astrocyte-conditioned medium (Bertin et al. 2012; Leone et al. 2006; Ormel et al. 2020), extracellular matrix (Sellgren et al. 2017), or serum-free medium (Etemad et al. 2012; Ohgidani et al. 2014; Rawat and Spector 2017; Ryan et al. 2017) in the presence of human recombinant cytokines (such as GM-CSF, M-CSF, NGF-β, CCL2, TGF-β, IFN-γ, and IL-34) that have been identified to drive microglia development and survival (Butovsky et al. 2014; Kierdorf et al. 2013).

In addition to MDMi, recent technological advancements with induced pluripotent stem cells (iPSCs) have led to the generation of several protocols to differentiate iPSCs into representative microglial cells (iPSC-MG) (Haenseler and Rajendran 2019; Hasselmann and Blurton-Jones 2020; Speicher et al. 2019). First, iPSCs are induced to a hematopoietic lineage and into erythro-myeloid or hematopoietic progenitors via stimulation with simple growth factor cocktails (minimally BMP4, VEGF, SCF, followed by IL-3, M-CSF). Subsequently, microglial differentiation is achieved either by co-culture with neural cells (Haenseler et al. 2017; Muffat et al. 2016; Pandya et al. 2017; Takata et al. 2017), by application of neural precursor conditioned media (Banerjee et al. 2020), or by addition of cytokines secreted from neurons and astrocytes to the culture media to mimic their presence (Abud et al. 2017; Douvaras et al. 2017; McQuade et al. 2018; Muffat et al. 2016).

As it is becoming evident that the phenotype of microglia is dependent on the CNS environment, co-culture with astrocytes and/or neurons and 3D culture systems have been shown to further induce the maturation of microglia. Cerebral organoids recapitulate many structural, developmental, and functional features of the human brain, including cytoarchitecture, cell diversity, and transcriptional profile (Chan et al. 2021; Chiaradia and Lancaster 2020; Qian et al. 2019; Sidhaye and Knoblich 2021). The generation of cerebral organoids can be divided in two categories: non-patterned or patterned. Non-patterned organoids are spontaneously differentiated from iPSCs via endogenous patterning cues and self-organize into various brain regions, ranging from the retina to hindbrain. Alternatively, patterned organoids are differentiated into one specific brain region through the addition of exogenous signaling molecules and growth factors to induce iPSC differentiation towards the desired lineage (Qian et al. 2019; Sidhaye and Knoblich 2021; Kim et al. 2021). A potential limitation of patterned cerebral organoids is the inhibition of mesoderm and endoderm formation including the cells derived from these germ layers in particular microglia. Recently, Ormel and colleagues reported the innate development of microglia, referred to as organoid-derived microglia (oMG, within unpatterned cerebral organoids (Ormel et al. 2018). Other CNS-microglia co- and tri-culture approaches include co-culturing iPSC-derived microglia with cerebral organoids (Abreu et al. 2018; Abud et al. 2017; Brownjohn et al. 2018; dos Reis et al. 2020; Lin et al. 2018; Muffat et al. 2016), or adding iPSC-derived astrocytes and neurons to iPSC-derived microglia (Haenseler et al. 2017; Park et al. 2018; Ryan et al. 2020; Takata et al. 2017).

### Comparison of microglia morphology

**Fig. 1:**
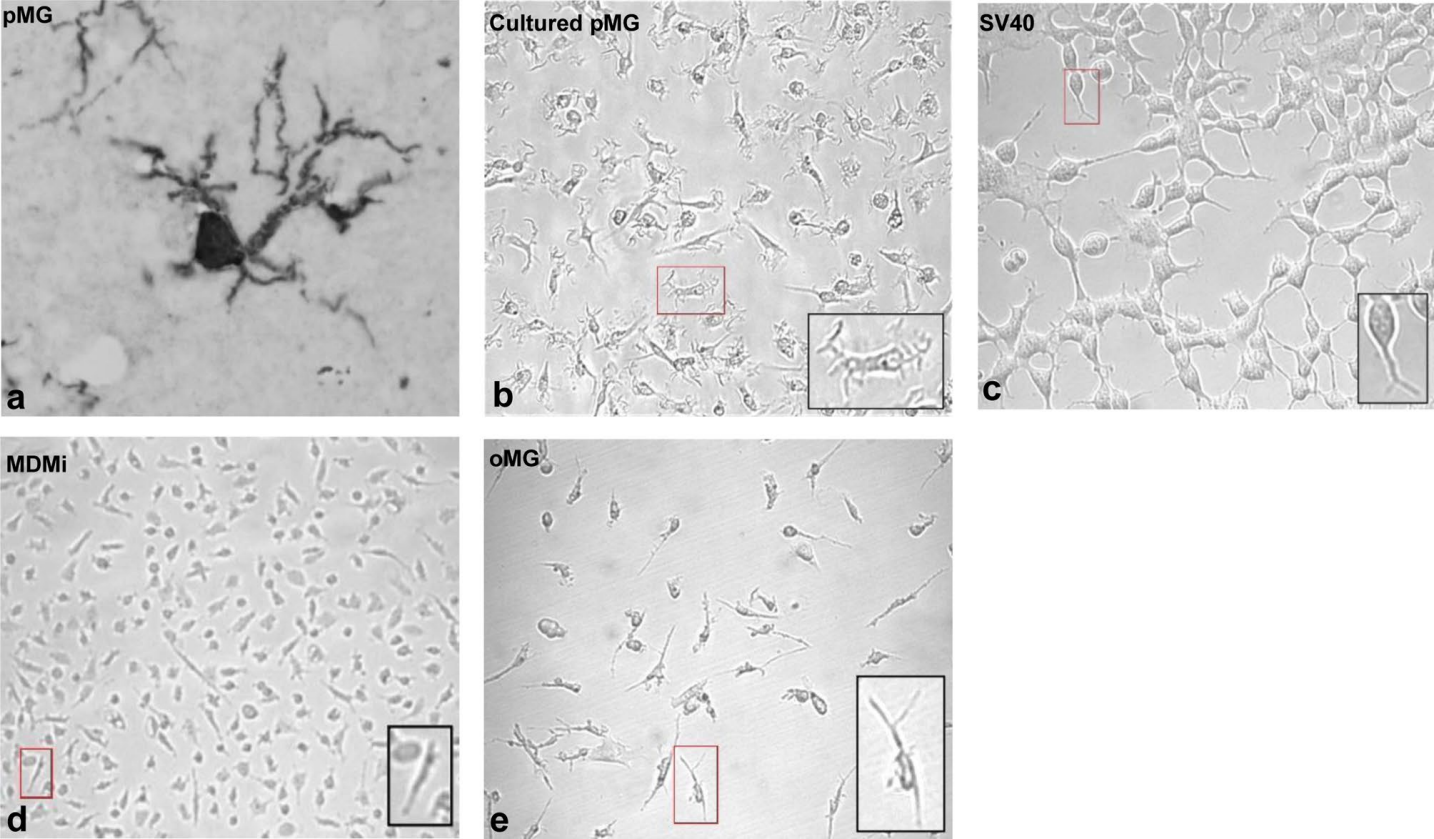
Morphology of human primary microglia and four different microglial in vitro culture models. **a** IBA-1 stained microglia in DAB-stained human brain sections of GFM at 40 × magnification. **b–e** Phase-contrast images of microglial culture models: adult primary microglia at 7 days post isolation **b**, SV40 microglial cell line **c**, monocyte-derived microglia **d**, and organoid-derived microglia **e**. Magnification = 10 × **d** and 20 × **b, c, e**

In the human adult brain, a variety of morphological forms is seen, with differences between gray and white matter (Geirsdottir et al. 2019; Salamanca et al. 2019; Torres-Platas et al. 2014). A so-called ramified morphology is classically assigned to be specific to microglia. This ramified morphology is one of the main characteristics used as a read-out parameter to optimize microglia in vitro culture models. A ramified morphology refers to cells with a small soma and very long and fine arborized processes, with primary, secondary, tertiary, and even quaternary branches (Walker et al. 2014) (Fig. 1a). These processes are highly dynamic and used for the active surveillance of the surroundings of the cells (Davalos et al. 2005; Nimmerjahn et al. 2005). Pathological events in the CNS, such as an infection, bleeding, hypoxia, cell death, and neurodegeneration, lead to activation of microglia, which is accompanied by a rapid change in microglia morphology (Wolf et al. 2017). In the first stage, the processes of the microglia are shortened and widened, a morphology referred to as reactive microglia. In the next stage, the soma of the microglia is enlarged with no or very few processes. This stage is referred to as an amoeboid morphology (Davalos et al. 2005; Nimmerjahn et al. 2005; Stence et al. 2001).

Following brain isolation, fetal and adult microglia show an amoeboid morphology with no or few processes. After a few days in culture, the cells will develop processes with some ramifications but these processes are never as arborized and complex as the microglia in situ (Levtova et al. 2017; Lue et al. 2019; Mizee et al. 2017; Tewari et al. 2021) (Fig. 1b). This disparity in morphology is evident across every in vitro culture model (Table 1). Commercially available human microglial cells lines SV40 and HMC3 have a varied morphology of globular and spindle-shaped cells with short processes and a few primary branches that fuse together when confluence is reached (Akhter et al. 2021; Chiavari et al. 2019; Dello Russo et al. 2018) (Fig. 1c). Similar morphology was observed in the novel microglial cell line hµglia clone 20 (C20) (Garcia-Mesa et al. 2017). Initial monocyte differentiation protocols generated MDMi with an elongated or small cellular body and a few unbranched processes; however, further optimization of the differentiation medium, e.g., the addition of IL-34 and GM-CSF, generated MDMI with a round or ovoid cell body with several primary processes with primary and a few secondary branches (Bertin et al. 2012; Leone et al. 2006; Ohgidani et al. 2014; Ryan et al. 2017; Sellgren et al. 2017) (Fig. 1d).

**Table 1:** Overview of morphology, phagocytosis, and inflammatory responsiveness of microglial in vitro culture models

Culture model	Difficulty	Culture days to microglia	Coating	Morphology	Function
Adult primary microglia	Intermediate	0	Poly-l-lysine, none	Spindle shape with a few processes and some 1st-degree branching	Phagocytosis: pHrodo-labeled myelin, fluorescent beads Inflammation response to: IL-1β, LPS, IFNγ, dexamethasone, IL-4
Human microglial cell lines	Easy	0	None	Globular and spindle-shaped cells with short processes	Phagocytosis: pHrodo-labeled synaptosomes, live neural progenitor cells, dead neuronal cells, Aβ42 Inflammation response to TNF-α, IL-1β, IFNγ, LPS
Monocyte-derived microglia	Easy	10–16	Poly-l-lysine, Geltrex, none	Round or ovoid cell body with several processes with 1st-degree branching	Phagocytosis: iC3b-coated beads, pHrodo-labeled synaptosomes, live neural progenitor cells, fluorescent-labeled *S. aureus*, latex beads, zymosan particles Inflammation response to LPS, IL-6, dexamethasone
iPSC-derived microglia	Intermediate	25–74	CellBIND, Primaria, Matrigel, poly-l-ornithine, fibronectin, gelatin, none	Amoeboid shaped cell body with several 1st- and 2nd-degree branched processes	Phagocytosis: zymosan-coated microbeads, *Escherichia coli*/*S. aureus* particles, fibrillar beta-amyloid, tau oligomers, and synaptosomes Inflammation response to IL-1β, IFNγ, LPS
Organoid-derived microglia	High	> 31	Matrigel embedment	Spindle-shaped cells with several processes with 1st-and 2nd-degree fine spines	Phagocytosis: iC3b-coated beads Inflammation response to LPS, dexamethasone

Out of all the models, iPSC-MG and oMG have the closest resemblance to the morphology of microglia in situ. iPSC-MG cultures consist mainly of amoeboid-shaped cell bodies from which several primary processes emerge with secondary and a few tertiary branches (Abud et al. 2017; Banerjee et al. 2020; Brownjohn et al. 2018; Douvaras et al. 2017; Muffat et al. 2016; Takata et al. 2017). oMG exhibits an elongated or amoeboid-shaped cell body with a few secondary branched processes (Ormel et al. 2018). Upon isolation and culture, oMG has a spindle shape with a few processes from which some 2nd-degree fine spines emerge (Ormel et al. 2018) (Fig. 1e).

### Comparison of microglia immune function

Under normal physiological conditions, microglia control neuronal viability, phagocytose degenerating neurons, remove excessive synaptic elements, and guide angiogenesis to support the establishment of functional neural circuits (Kierdorf and Prinz 2017; Wolf et al. 2017). Under pathological conditions, microglia secrete a broad spectrum of cytokines, chemokines, reactive oxygen species, and neurotrophic factors to promote and/or control inflammation and phagocytose apoptotic cells and cellular and myelin debris (Colonna and Butovsky 2017; Galloway et al. 2019; Rodríguez-Gómez et al. 2020). However, these immune functions are shared with other myeloid cells in the CNS including non-parenchymal macrophages and infiltrating macrophages from the periphery (Abe et al. 2020; Li and Barres 2018). Several studies reported that microglia and macrophages have different functions during or following injury (Evans et al. 2014; Greenhalgh and David 2014; Plemel et al. 2020; Ritzel et al. 2015; Yamasaki et al. 2014). However, as microglia lose their ramified morphology in vitro and transform into an amoeboid phenotype in response to injury in vivo, it becomes increasingly difficult to discriminate between them. Consequently, it has been difficult to assign how microglia are different from other myeloid cells in terms of function.

Though not exclusive to microglia, we assessed two of the primary functions of microglia in each microglial model, namely the ability to phagocytose and the ability to induce an inflammatory response (Table 1). Each culture model reported to have phagocytic capabilities ranging from engulfment of zymosan- or iC3b-coated microbeads to more CNS-relevant substrates such as synaptosomes, neural progenitor cells, apoptotic neurons, and myelin (Abud et al. 2017; Amos et al. 2017; Banerjee et al. 2020; Douvaras et al. 2017; Garcia-Mesa et al. 2017; Hendrickx et al. 2014; McQuade et al. 2018; Mizee et al. 2017; Ormel et al. 2018; Pandya et al. 2017; Rawat and Spector 2017; Sellgren et al. 2017; Takata et al. 2017). Direct comparison to cultured pMG revealed a similar phagocytic ability with MDMi and oMG, whereas SV40 had limited phagocytosis (Ormel et al. 2018; Rawat and Spector 2017; Sellgren et al. 2017).

Like phagocytosis, the inflammatory responses of microglial culture models are validated using a variety of pro- and anti-inflammatory stimuli and read-out parameters (Table 1). Stimulation of cultured pMG with the pro-inflammatory stimulus LPS (lipopolysaccharide) classically leads to activation of microglia with increased secretion of IL-6, IL-1β, and TNF-α (Lee et al. 2002; Melief et al. 2012, 2016; Nagai et al. 2001; Rustenhoven et al. 2016). To compare the models with each other, secretion of IL-6, IL-1β, and TNF-α was assessed and compared to cultured pMG post stimulation with LPS. LPS stimulation of SV40, iPSC-MG, and oMG led to high induction of TNF-α release, whereas the secretion of IL-6 and IL-1β varied from modest and moderate in the cell lines MDMi and iPSC-MG to significant in oMG and cerebral organoids (Abreu et al. 2018; Abud et al. 2017; Banerjee et al. 2020; Ormel et al. 2018, 2020; Pandya et al. 2017; Patel et al. 2016). Direct comparison to cultured pMG revealed a significantly higher inflammatory response (IL-6 and IL-1β) in oMG (Ormel et al. 2018).

As a whole, each microglial in vitro culture model is capable of phagocytosis and showed varying degrees of LPS responsiveness. However, as previously mentioned, these functions are not exclusive to microglia but shared with other myeloid cells such as macrophages. Therefore, to further investigate the microglial phenotype of the culture models, we compared the transcriptome of the cultured pMG, the MDMi, the iPSC-MG, and the oMG to the transcriptome of uncultured adult pMG.

### Gene expression

Markers that have classically been used to identify microglia in brain tissue, such as HLA-DR and CD68, are also expressed by other myeloid cells and can therefore not be used to determine whether a cell model reflects microglia or another myeloid cell type. Recent transcriptomic studies have compared human microglia with other myeloid cells (Böttcher et al. 2019; Gosselin et al. 2017; Kracht et al. 2020; Ormel et al. 2018) and identified a specific gene signature for adult and fetal microglia. This includes markers such as *AIF1, TMEM119, P2RY12, CX3CR1, CSF1R*, and *TREM2* (Bennett et al. 2016; Butovsky et al. 2014; DePaula-Silva et al. 2019; Galatro et al. 2017; Gautiar et al. 2012; Gosselin et al. 2017; Haage et al. 2019; Hickman et al. 2013; Konishi et al. 2017; Mildner et al. 2017; Ormel et al. 2018) (Table 2).

**Table 2:** Overview of microglia-enriched markers

Marker (gene)	Name	Cell type	Function	Reference
TMEM119	Transmembrane Protein 119	Microglia	Uncertain	Bennett et al. (2016), Satoh et al. (2016)
P2RY12	Purinergic Receptor P2Y12	Microglia	Purinergic receptor required for microglia chemotaxis in response to CNS injury	Bennett et al. (2016), Butovsky et al. (2014)
CSF1R	Colony Stimulating Factor 1 Receptor	Microglia and other myeloid lineage cells	Cell surface receptor that directly controls the development, survival, and maintenance of microglia and plays a pivotal role in neuroinflammation	Chitu et al. (2016), Erblich et al. (2011), Nandi et al. (2012)
CX3CR1	C-X3-C motif chemokine receptor 1	Microglia and other myeloid lineage cells	Chemokine receptor critical in controlling microglia numbers, synaptic pruning, and functional brain connectivity	Jones et al. (2010)
TREM2	Triggering Receptor Expressed on Myeloid Cells 2	Microglia and other myeloid lineage cells	Regulatory protein involved in microglia activation and phagocytosis of apoptotic neurons	Colonna (2003), Neumann and Takahashi (2007)
AIF1	Ionized Calcium-Binding Adapter Molecule 1	Microglia and other myeloid lineage cells	Cytoplasmic protein involved in microglia motility, membrane reorganization and phagocytosis	Imai et al. (1996), Sasaki et al. (2001)

Following isolation, cultured adult pMG lose part of the microglia-specific gene signature, with a downregulation of *P2RY12*, *TREM2*, and *TMEM119*, as well as an upregulation of inflammatory- and stress response-associated genes (Bohlen et al. 2017; Gosselin et al. 2017). This mature microglia signature, therefore, seems to be much dependent on the CNS environment (Gosselin et al. 2017). Accordingly, lower expression levels of *CX3CR1*, *P2RY12*, and *TMEM119* were generally reported in the microglial cell lines (Melief et al. 2016; Rai et al. 2020), MDMi (Melief et al. 2016; Ormel et al. 2020; Rai et al. 2020; Rawat and Spector 2017), and oMG (Ormel et al. 2018) compared to those in adult pMG.

To further characterize the transcriptomic similarity of microglial culture models to adult pMG, we compared previously published RNA-seq data of cultured adult pMG (Gosselin et al. 2017; Lopes et al. 2020), fetal pMG (Douvaras et al. 2017), iPSC-MG (Brownjohn et al. 2018; Douvaras et al. 2017), MDMi (Ormel et al. 2020), monocytes (Gosselin et al. 2017; Ormel et al. 2020), and oMG (Ormel et al. 2018) to that of uncultured adult pMG (Gosselin et al. 2017; Ormel et al. 2018, 2020) leveraging (i) Pearson correlation, (ii) principal component analysis (PCA), and (iii) unsupervised hierarchical clustering.

#### Comparison of the full transcriptome

**Fig. 2:**
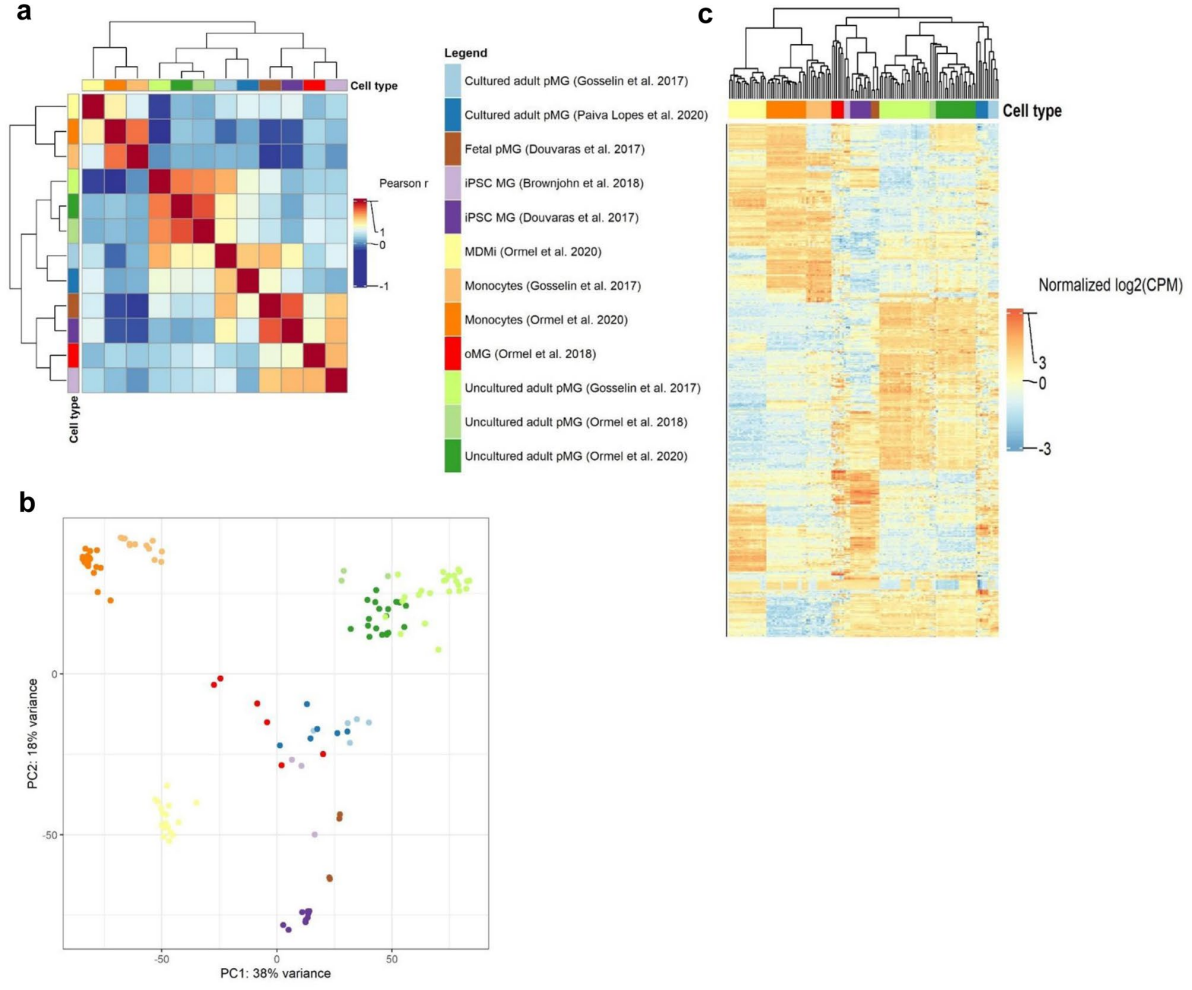
Gene expression analysis of microglial culture models on the 500 most variable genes. Legend shows color coding for cell type. **a** Heatmap depicting the Pearson *r* correlation effect sizes between cell types based on the 500 most variable genes. Clustering dendrogram is based on Euclidean distances. **b** PC plot depicting cell-type distances based on expression variance in the 500 most variable genes. Clustering dendrogram is based on Euclidean distances. **c** Heatmap of log2(CPM) expression values for the 500 most variable genes depicted for each cell type

**Fig. 3:**
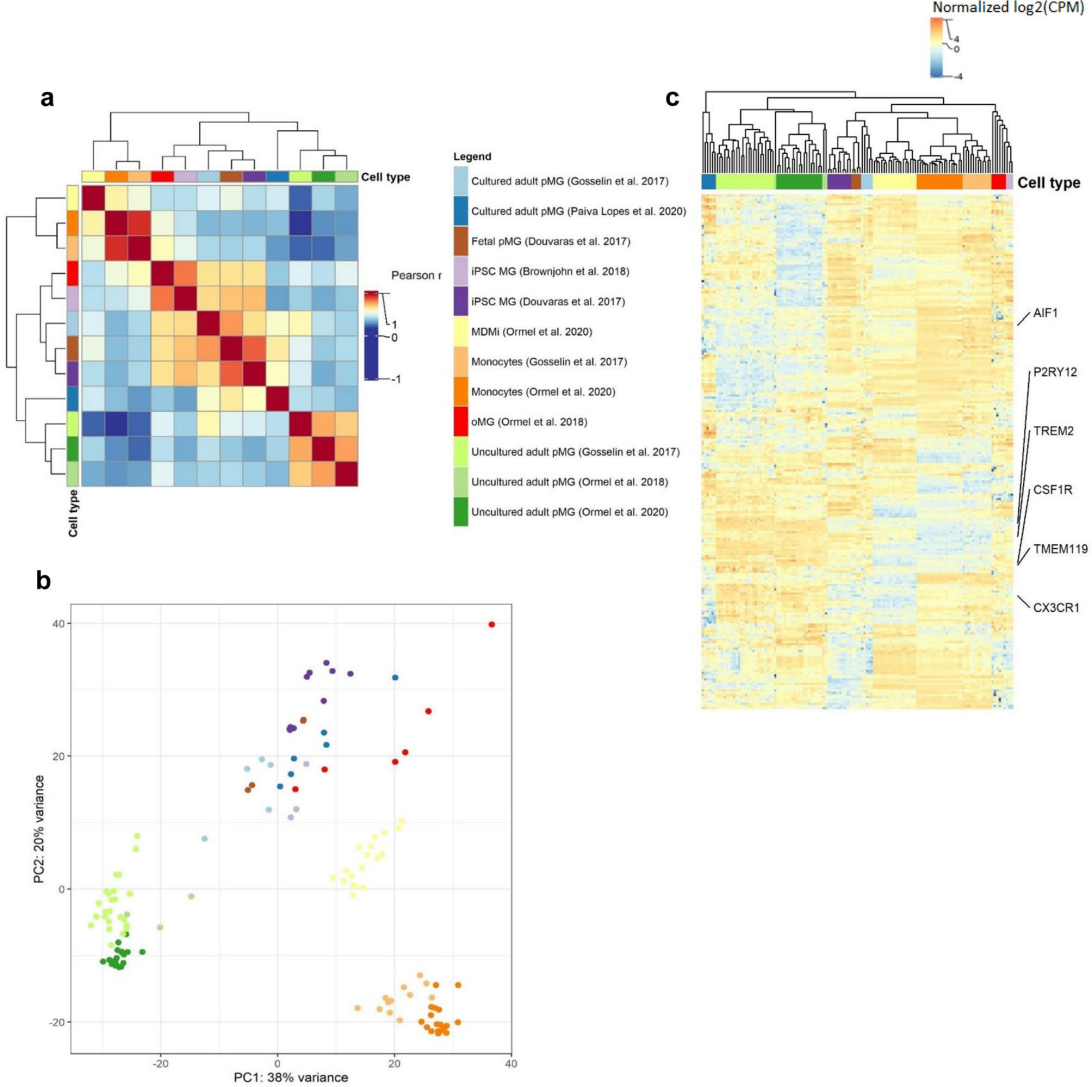
Gene expression analysis of microglial culture models on a microglia-specific core signature. **a** Heatmap depicting Pearson *r* correlation effect sizes between the cell types based on microglia core gene expression. Clustering dendrogram is based on Euclidean distances. Clustering dendrogram depicts Euclidean distances. **b** PC plot depicting cell-type similarities based on expression variance within microglia core genes. **c** Heatmap of log2(CPM) values for microglia core genes extracted from Patir et al. (2019)

To examine the general relationship between the gene expression of the various culture models, we correlated the counts of a collapsed version of the three uncultured adult pMG datasets with the counts of the cultured microglial model datasets. The regression coefficients of the full transcriptome datasets of uncultured adult pMG and the different culture models were all high, ranging from 0.91 for the cultured pMG, to 0.71 for the oMG (Fig. S1). The results of our principal component, correlation, and unsupervised clustering analyses of cluster-defining and microglia genes show a high similarity of same cell-type samples despite their origin from different studies, supporting the validity of our comparative analyses (Figs. 2 and 3).

To thoroughly understand the similarities and dissimilarities of the transcriptomic architecture of the culture models to uncultured adult pMG, we then decided to shift the perspective towards a more specific examination of our RNA-seq data by performing our analyses on two selected gene sets: (i) the 500 most variable genes (i.e., the genes that are most distinguishing across samples in terms of expression) and (ii) a microglia-specific core signature recently defined by Patir et al. (2019).

#### Most variable genes

Leveraging the most variable genes only (i.e., those most defining of inter-sample difference), we found that datasets of the same model system were moderately (*r* > 0.6; iPSC-MG, cultured adult pMG) to strongly correlated (*r* > 0.7*;* uncultured adult pMG, monocytes), presenting evidence for the validity of our comparisons (Fig. 2a).

Only the cultured adult pMG by Gosselin et al. (2017) were moderately correlated with uncultured adult pMG (*r* > 0.4). These results were mirrored in the inter-sample distances we observed on our PCA based on the 500 most variable genes: samples of monocytes or uncultured adult pMG across different datasets clustered together, respectively (suggesting high intra-cell-type similarity), while the cultured model systems, MDMi disregarded, formed an interspersed cluster apart from uncultured adult pMG and monocytes (Fig. 2b). To further extract information about transcriptomic cell-type similarity, we interrogated the data on hierarchical clustering (Fig. 2c). Again, we found small intra-cell-type sample distances where samples of the same cell type but different datasets corresponded to the same clusters. In line with our correlation results, the cultured adult pMG samples also clustered more proximal to the uncultured adult pMG samples than would our PCA suggest, further emphasizing the similarity of these model systems. These results corresponded to those of previously conducted comparative analyses (Ormel et al. 2018).

#### Expression of microglia core genes

Next, we aimed to examine the transcriptomic similarity of cell types by repeating our analyses in the context of a microglia-specific core signature of 249 genes defined by Patir et al. (2019). Again, we found moderate (*r* > 0.6; iPSC-MG, cultured pMG) to strong (*r* > 0.8: uncultured adult pMG, monocytes) intra-cell-type sample correlations across datasets (Fig. 3a). Opposed to earlier results, cultured pMG now were correlated only moderately with uncultured adult pMG (*r* > 0.6), followed by oMG and MDMi (*r* > 0.5). Similarly, the PCA displayed that inter-sample distances for the uncultured adult pMG and monocyte datasets were diminishingly small within cell types but distinguishably large between cell types (Fig. 3b). MDMi samples now showed a smaller distance to samples of microglial culture models while these remained an interspersed cluster on their own. The hierarchical clustering analysis as well underlined the internal consistency regarding intra-cell-type sample distances (Fig. 3c). Contrary to the inter-sample distances on our PCA, MDMi clustered closer to monocytes which together formed a cluster separate from the microglial culture model samples. Interestingly, oMG samples represented, together with the iPSC-MG by Brownjohn et al. (2018), a cluster distinct from all other cell types. These observations, however, aligned with the results of our correlation analysis. This suggests that the examined model systems break down in reflecting the microglia phenotype on a transcriptomic level when focusing on microglia-specific expression programs. In line with previous transcriptomic studies, microglia-specific genes including *TMEM119, P2RY12, CX3CR1*, and *CSF1R* were low in the in vitro culture models compared to uncultured adult pMG. Interestingly, adult pMG kept in culture for 7 and 10 days had a higher expression of *CSF1R* and *TMEM119* compared to uncultured adult pMG. Whether this is due to prolonged culture or a response to cell death and/or cell debris remains to be determined in future studies (Fig. S2)*.* MDMi and iPSC-MG also had a higher expression of *IBA1* and *TREM2* compared to cultured adult pMG.

#### HIV-relevant gene expression

**Fig. 4:**
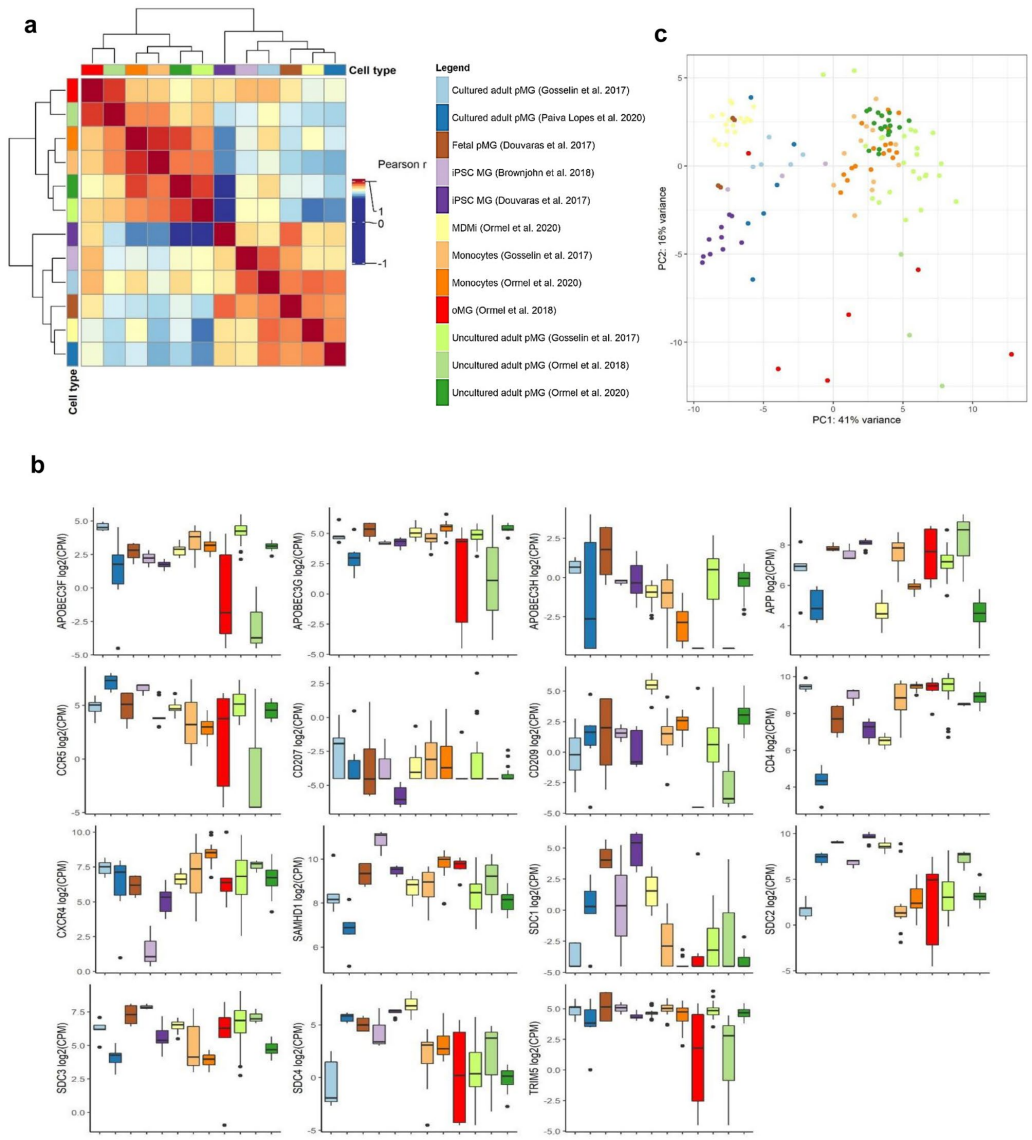
Gene expression analysis of microglia culture models on HIV-relevant genes. **a** Heatmap of Pearson *r* for between each cell type (cluster distances are Euclidean). **b** Boxplot of log2(CPM) for selected HIV genes. **c** PC plot depicting cell-type distances based on expression variance within selected HIV genes

To identify microglial culture models that are suitable for HIV research, we evaluated the similarity of the culture models with uncultured adult pMG at the level of HIV-relevant genes (Fig. 4). Undoubtedly, a good HIV microglia model must express the CD4 receptor and CCR5 co-receptor ideally at levels similar to microglia in situ. *CD4* gene expression in uncultured adult pMG was similar to oMG, monocytes, cultured pMG from Gosselin et al. (2017) and iPSC-MG from Brownjohn et al. (2018), but considerably higher than MDMi, cultured pMG from Lopes et al. (2020), and iPSC-MG from Douvaras et al. (2017) (Fig. 4b). Opposite to *CD4*, expression of *CCR5* in the culture models was generally similar to that in uncultured pMG; however, the uncultured adult pMG from Ormel et al. (2018) had considerably lower expression compared to the other uncultured adult pMG datasets. Although the viruses found in the CNS predominantly use CCR5, we also analyzed the expression of the CXCR4 co-receptor. In general, *CXCR4* expression in the models was similar to that in uncultured adult pMG, except for the lower expression seen in iPSC-MG. Besides the major HIV receptors, we also assessed the expression levels of several restriction factors that were found to restrict HIV infection, replication, and/or spread. *TRIM5* and *APOBEC3G* expression in the culture models was similar to that in uncultured adult pMG, although oMG had a lower *TRIM5* expression compared to the other models. Expression of *SAMHD1* was lower in cultured adult pMG (Lopes et al. 2020) and higher in iPSC-MG (Brownjohn et al. 2018) compared to that in uncultured adult pMG.

Looking at overall model similarity, strikingly, Pearson correlation coefficients were very high (*r* > 0.8) among MDMi and cultured pMG, as well as among monocytes, uncultured adult pMG, and oMG, respectively (Fig. 4b). Unlike before, cell-type-specific clusters of samples started to diffuse and became less apparent. That is, on our PCA the distances between monocytes and uncultured adult pMG diminished, MDMi clustered with the highly interspersed cultured adult pMG, and oMG now scattered across the first two PCs (Fig. 4c). This suggests that the HIV-relevant gene expression profile is not specific to one model. Hierarchical clustering of samples supported the strong notion of diffusion but provided a higher conservation of the same-cell-type sample cluster identity. Notably, in all three analyses, monocytes displayed the highest degree of similarity to uncultured adult pMG in terms of the examined HIV gene expression, suggesting high conservation of HIV gene expression compared to uncultured adult pMG. CCR5 expression, however, was low.

## Comprehensive overview: HIV research on microglia models

### Overview of HIV DNA and RNA in microglia of human brain tissues

That microglia are a site of HIV infection in the CNS is supported by the detection of (integrated) HIV DNA in microglia of HIV-positive individuals by laser capture microdissection coupled with polymerase chain reaction (PCR) (Churchill et al. 2006; Thompson et al. 2011; Trillo-Pazos et al. 2003). Churchill and colleagues detected integrated HIV DNA in the isolated CD68 + microglial/macrophage cells in all 3 examined HIV-positive individuals that died with HIV-associated dementia (Churchill et al. 2006). Trillo-Pazos and colleagues also consistently detected HIV DNA in microdissected brain tissue from all four HIV + individuals (2 pediatric and 2 adult patients) with HIV encephalitis (HIVE) (Trillo-Pazos et al. 2003). HIV DNA levels were quantified in duplicate pools of 100 CD68 + microglia/macrophage cells, and extrapolation to a standard curve revealed that about 1–10% of the investigated cells are likely to harbor HIV DNA. HIV *gag* DNA was more prominent in cases with notable microgliosis (Trillo-Pazos et al. 2003). Another study by Thompson and colleagues detected HIV DNA in both HIV-positive encephalitic patients, with evident microglia activation and/or microglial nodules, and in 4 of the 5 HIV-positive presymptomatic individuals who died before pathological evidence of HIVE (Thompson et al. 2011). HIV DNA levels were quantified in pools of 200 CD68 + parenchymal microglial cells (distinguished by shape and location from the perivascular macrophages), and 10% of the analyzed replicates were positive by triple-nested PCR in both symptomatic patients and the majority of the presymptomatic patients (Thompson et al. 2011).

However, these post-mortem studies do not reflect modern-day combined antiretroviral therapy (cART) patients with effective suppressive therapy. Lamers and colleagues measured HIV DNA in tissues of 20 virally suppressed HIV + individuals by real-time PCR and droplet digital PCR (ddPCR) and found HIV DNA in 48 of 87 brain tissues (Lamers et al. 2016). A more in-depth cellular analysis was done by Tso and colleagues, who detected HIV RNA and/or DNA within CD68 + cells in 3 out of 4 virally suppressed individuals infected with HIV subtype C using ddPCR and RNA/DNA scope ISH (Tso et al. 2018). The distribution of the HIV viral genome was proposed to most likely be the result of a random event as no obvious distribution pattern was observed among the various brain compartments (frontal lobe, cerebellum, hippocampus, basal ganglia, temporal lobe, parietal lobe, and occipital lobe) (Tso et al. 2018). This being said, viral strain compartmentalization has been found between different brain regions (frontal lobe, occipital lobe, and parietal lobe) (Brese et al. 2018).

A recent study by Ko and colleagues confirmed the persistence of HIV DNA in virally suppressed patients with (*n* = 8) and without (*n* = 8) HAND using a highly specific DNAscope in situ hybridization technique (Ko et al. 2019). In all 16 cases, HIV DNA was found exclusively in CD68 + microglia/perivascular macrophages. Small clusters of isolated HIV RNA signals, which were infrequent and very focal, were observed in a small group (*n* = 6) of virally suppressed patients with (*n* = 3) and without HAND (*n* = 3) (Ko et al. 2019). The evidence of low copies of HIV RNA in some cases suggests either spontaneous viral reactivation or ongoing low level replication despite suppressive cART (Ko et al. 2019; Tso et al. 2018).

### Overview of HIV infection in cultured microglia models (Table 3)

**Table 3:** Overview of the characteristics of all the microglial in vitro culture models

Microglia model	Name	Co-culture	Microglia markers		HIV research citations (*n*)^a^	Reference
Primary microglia	Primary microglia		CD11b, CD45, TMEM119, CD68, HLA-DR, P2RY12, CX3CR1, PU.1		**(13)** Albright et al. (1999), Asahchop et al. (2017), Castellano et al. (2017), Cenker et al. (2017), Garcia-Mesa et al. (2017), Ghorpade et al. (1998), Huang et al. (2011), Lee et al. (1993), Mamik and Ghorpade (2014), Schuenke and Gelman (2003), Strizki et al. (1996), Tatro et al. (2014), Zenón et al. (2015)	Mizee et al. (2017), Olah et al. (2012), Rustenhoven et al. (2016), Zhang et al. (2016)
Human microglia cell lines	SV40		IBA1, TREM2, CD11b, CD68			Chiavari et al. (2019)
	hµglia		CD68, P2RY12, CD11b		**(4)** Alvarez-Carbonell et al. (2017), Garcia-Mesa et al. (2017), Ingram et al. (2020), Rai et al. (2020)	Garcia-Mesa et al. (2017)
	HMC3, CHME3/5, C13NJ		CD68, CD11b, CD45, IBA1, CX3CR1		**(18)** Ambrosius et al. (2017), Campbell et al. (2017, 2019), Chai et al. (2017), Chugh et al. (2007), Delaney et al. (2017), dos Reis et al. (2020), Francis et al. (2020), Ingram et al. (2020), Jadhav et al. (2014), Lisi et al. (2016), Malikov and Naghavi (2017), Mishra et al. (2012), Rai et al. (2020), Samikkannu et al. (2016), Tomitaka et al. (2018), Wires et al. (2012), Zenón et al. (2015)	Dello Russo et al. (2018), Janabi et al. (1995)
Monocyte-derived microglia	MDMi		HLA-DR, IBA1		**(2)** Cherrier et al. (2011), Leone et al. (2006)	Leone et al. (2006)
	iMG		CX3CR1, HLA-DR, CD45			Ohgidani et al. (2014)
	iMG		TMEM119, P2RY12, PU.1			Sellgren et al. (2017, 2019)
	MMG		IBA1, CD11b, CD45, HLADR_LOW_, P2RY12, CD68		**(2)** Rawat et al. (2019), Rawat and Spector (2017)	Rawat and Spector (2017)
	ML	Astrocytes	CD11b, TREM2, IBA1			Noto et al. (2014)
	MDMi		IBA1		**(1)** Bertin et al. (2012)	Bertin et al. (2012)
	MDMi		P2RY12, CSF1R, TREM2		**(1)** Rai et al. (2020)	Ryan et al. (2017)
	iMG		TREM2, HLADR			Ormel et al. (2020)
	M-MG		CX3CR1			Etemad et al. (2012)
iPSC-derived microglia	pMGLs			CD45, IBA1, P2RY12, TMEM119		Muffat et al. (2016)
	iPSC-MG			CD11b, CX3CR1, IBA1, P2RY12, TMEM119		Douvaras et al. (2017)
	iMGLs			CD45, CX3CR1, P2RY12, TREM2, PU.1, CSF1R, CD11b		Abud et al. (2017)
	iMGLs			IBA1, TMEM119		Xu et al. (2019)
	iPSC-derived microglia			IBA1, CD45, TREM2		Brownjohn et al. (2018)
	Co-pMG	iPSC-derived neurons		CD11b, CD45, IBA1		Haenseler et al. (2017)
	iMicros	iPSC-derived neurons		IBA1		Takata et al. (2017)
	iPS-MG	Astrocytes		CD11b, CD45, CX3CR1, HLA-DR, IBA1, TREM2		Pandya et al. (2017)
	ScMglia			CX3CR1, P2RY12, TREM2, CSF1R, IBA1, CD11b		Amos et al. (2017)
	hiPSC-MG			CSF1R, P2RY12, TMEM119, TREM2, CX3CR1		Banerjee et al. (2020)
	iMg			CX3CR1, TMEM119, IBA1, P2RY12	**(1)** Ryan et al. (2020)	Ryan et al. (2020)
Cerebral organoids	Brain spheres + SV40 cell line	Neurons, astrocytes, oligodendrocytes		TMEM119, IBA1		Abreu et al. (2018)
	3D cortical organoids + iPSC-derived microglia	Neurons, astrocytes		ND		Brownjohn et al. (2018)
	3D BORG + iPSC-derived microglia	Neurons, astrocytes, oligodendrocytes		ND		Abud et al. (2017)
	hBORG + HMC3 cell line	Neurons, astrocytes		ND	**(1)** dos Reis et al. (2020)	dos Reis et al. (2020)
	Cerebral organoids	Neurons, astrocytes, oligodendrocytes		IBA1, CD68, CD11b, TREM2, CX3CR1, HLA-DR, CD45		Ormel et al. (2018)

*ND* not determined

^a^Research article of HIV infection studies with human lab strains

As mentioned earlier, viruses detected in the CNS are predominantly R5 M-tropic (Arrildt et al. 2015; Joseph et al. 2015; Joseph and Swanstrom 2018). Accordingly, primary microglia isolated from fetal and adult brain tissue in culture were shown to be primarily susceptible to HIV infection with R5 M-tropic HIV strains (HIV_ada_, HIV_Bal_, HIV_YU-2_, HIV_JRFL_, HIV_SF162_) (Albright et al. 1999, 2000; Asahchop et al. 2017; Castellano et al. 2017; Cenker et al. 2017; Garcia-Mesa et al. 2017; Ghorpade et al. 1998; Huang et al. 2011; Lee et al. 1993; Mamik and Ghorpade 2014; Schuenke and Gelman 2003; Strizki et al. 1996; Tatro et al. 2014; Zenón et al. 2015). It is noteworthy to mention that although every study observed HIV infection with an R5 M-tropic virus, different methods were used to isolate microglia that were subsequently grown in different culture media for 1 day up to 3 weeks before infection. As previously mentioned, this leads to significant changes in the microglial phenotype, which in turn could affect the susceptibility to HIV infection. This being said, cultured fetal and adult pMG seem to have a higher susceptibility to HIV infection compared to in vivo cells, with an infection rate of 40–50% at 72 h (Alvarez-Carbonell et al. 2019; Cenker et al. 2017), 75% at day 5 (Garcia-Mesa et al. 2017), and about 90% at day 6 post infection (Cenker et al. 2017). Moreover, HIV-infected microglia formed giant multinucleated syncytia that accumulated in the cultures over time and correlated with peaks in HIV capsid (p24) levels (Castellano et al. 2017; Cenker et al. 2017; Lee et al. 1993). This cytopathic effect is reminiscent of microglial nodular lesions observed in the brains of HIV-positive individuals with HAND and AIDS (Budka et al. 1987; Nebuloni et al. 2000), among which are the HIV-positive individuals with HIVE in the previously mentioned studies by Trillo-Pazos et al. (2003) and Thompson et al. (2011). Furthermore, addition of CCR5 inhibitor maraviroc on day 1 post infection blocked the increase in infection, indicating spread of HIV infection in these cultures (Cenker et al., 2017). Most laboratory-adapted T-cell tropic HIV strains require high surface density of the CD4 and CXCR4 receptor and hence were found to replicate inefficiently in human primary microglia (HIV_HxB2_, HIV_NL4-3_, HIV_LAI_) (Ghorpade et al. 1998; Strizki et al. 1996).

In line with cultured pMG, the HMC3 cell line, MDMi and iPSC-MG were reported to be susceptible to infection by several R5 M-tropic HIV strains (HIV_bal_, HIV_ada_, HIV_YU-2_, HIV_jago_) (Bertin et al. 2012; Chugh et al. 2007; Leone et al. 2006; Rai et al. 2020; Rawat and Spector 2017; Ryan et al. 2020; Samikkannu et al. 2016; Zenón et al. 2015). Zenón and colleagues directly compared HIV infection of cultured fetal pMG with infection of the microglial cell line HMC3 and showed tenfold higher levels of p24 production in the pMG cells as compared to HMC3 (Zenón et al. 2015). In contrast, Rai and colleagues could not detect infection of the C20 and HMC3 cells, which is in line with the fact that they were also unable to detect expression of the primary CD4 receptor in these cell lines (Rai et al. 2020). Rawat and Spector compared cultured fetal primary microglia with MDMi and showed similar HIV infection kinetics and indicated that after 20 days of infection the majority of cells were p24 positive (Rawat and Spector 2017). Productive HIV infection of MDMi is also shown in other in vitro studies (Bertin et al. 2012; Leone et al. 2006; Rai et al. 2020). Rai and colleagues directly compared iPSC-MG to MDMi and the C20 and HMC3 cell lines and showed productive infection in the iPSC-MG as well as in the MDMi (Rai et al. 2020). iPSC-MG infection resulted in virus levels that peaked at day 8 post infection and then declined. MDMi, in contrast, continued to produce virus over the 2-week experiment, albeit at about tenfold lower levels as seen in the iPSC-MG. Ryan and colleagues showed that iPSC-MG are highly susceptible to HIV infection with 95% of cells p24 positive after 15 days with extensive multinucleation exclusively associated with infection (Ryan et al. 2020). As observed for the pMG also, the HMC3 cell line (Chugh et al. 2007) and MDMi (Bertin et al. 2012) were found to be refractory to infection with the T-cell tropic HIV strain HIV_NL4-3_. To the best of our knowledge, there are no published studies of HIV research with organoid-derived microglia or 3D cerebral organoids in which microglia develop innately.

### Head-to-head comparison of HIV infection in microglial culture models

**Fig. 5:**
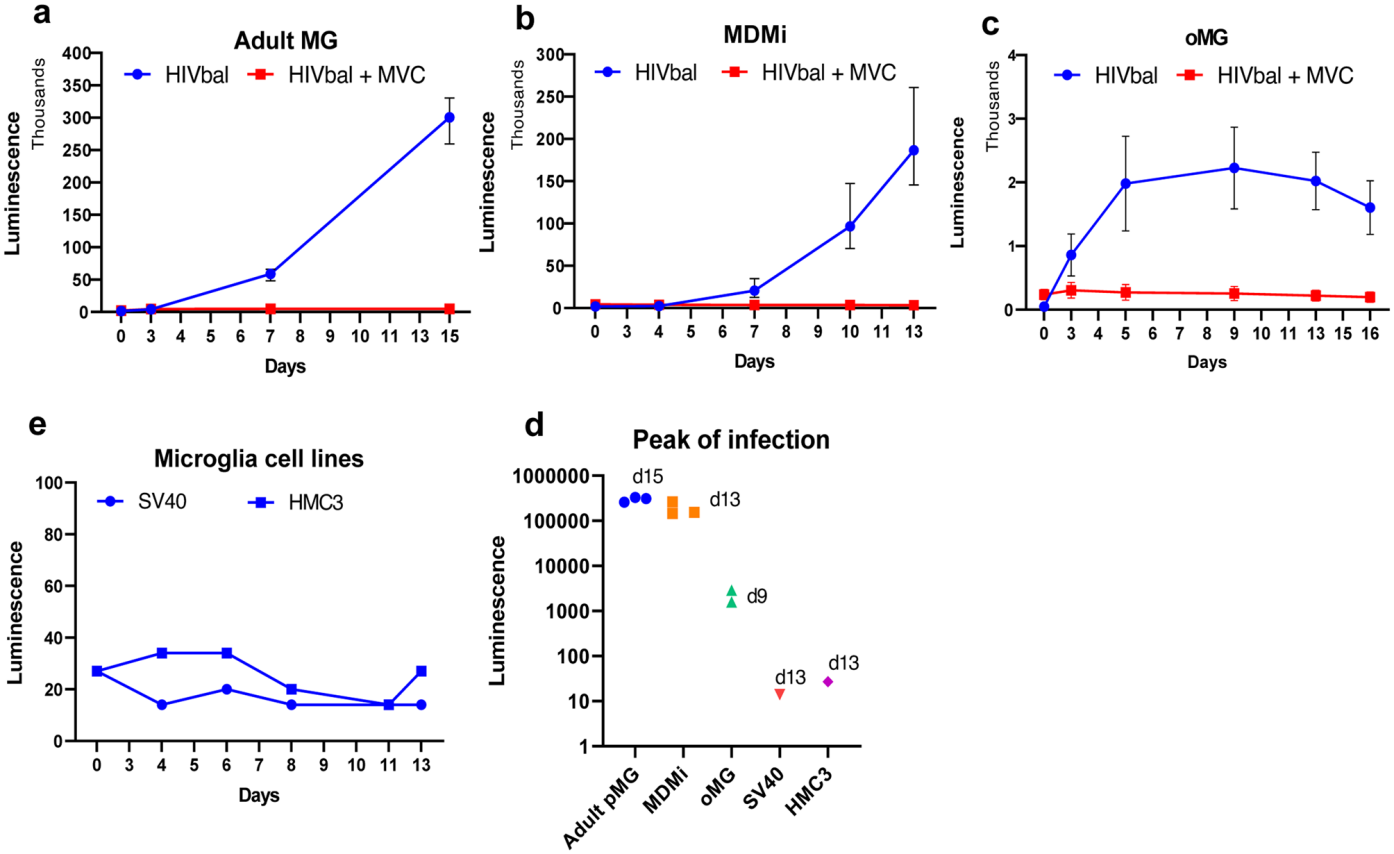
HIV infection and virus production in microglial culture models. Adult primary microglia **a**, MDMi **b**, oMG **c**, and microglial cell lines SV40 and HMC3 **e** were infected with 10 ng (p24 Gag) HIVbal with a luciferase tag and cultured for the indicated days. Supernatant was collected post infection on the indicated days, and virus production was measured with luminescence. **d** Peak infection day of each culture model

As previously mentioned, numerous studies have been published on the susceptibility of each culture model to HIV infection. However, there is a gap in literature in how the different microglial culture models compare with each other and most importantly to cultured pMG. Therefore, we performed a head-to-head comparison of the different microglial culture models by infecting each model, except for iPSC-MG, with the R5 M-tropic HIV strain HIV_bal_, equipped with a luciferase tag, under the same experimental conditions (Fig. 5). Extracellular virus production was measured indirectly by quantifying the release of luminescence over time.

As expected, cultured adult pMG were highly susceptible to HIV_bal_ infection and showed continuous virus production with a peak infection on day 15 (Fig. 5a). A similar infectivity and pattern was observed in MDMi that peaked on day 13 (Fig. 5b). To investigate whether oMG are susceptible to HIV infection, we isolated oMG innately developed within human cerebral organoids before HIV_bal_ infection. Organoid-derived microglia were susceptible to HIV_bal_ infection; however, contrary to cultured adult pMG and MDMi, peak infection was reached on day 9 and luminescence was also substantially lower (> 150-fold) in oMG compared to cultured adult pMG (Fig. 5c, d). Pre-incubation of microglia with the CCR5 inhibitor maraviroc successfully prevented viral infection in all culture models, indicating that infection occurred primarily through the CCR5 receptor. Microglial cell lines SV40 and HMC3 did not support the infection of HIV_bal_ (Fig. 5e).

Next, we also infected cultured pMG and MDMi with HIV_bal_ equipped with a GFP tag to evaluate the fraction of HIV-infected cells and the effect of HIV infection on cell morphology. GFP + microglia could be detected as early as 4 days post infection in both culture models, which increased to ~ 90–95% on day 15. GFP expression was also exclusively found in giant multinucleated cells (data not shown).

**Fig. 6:**
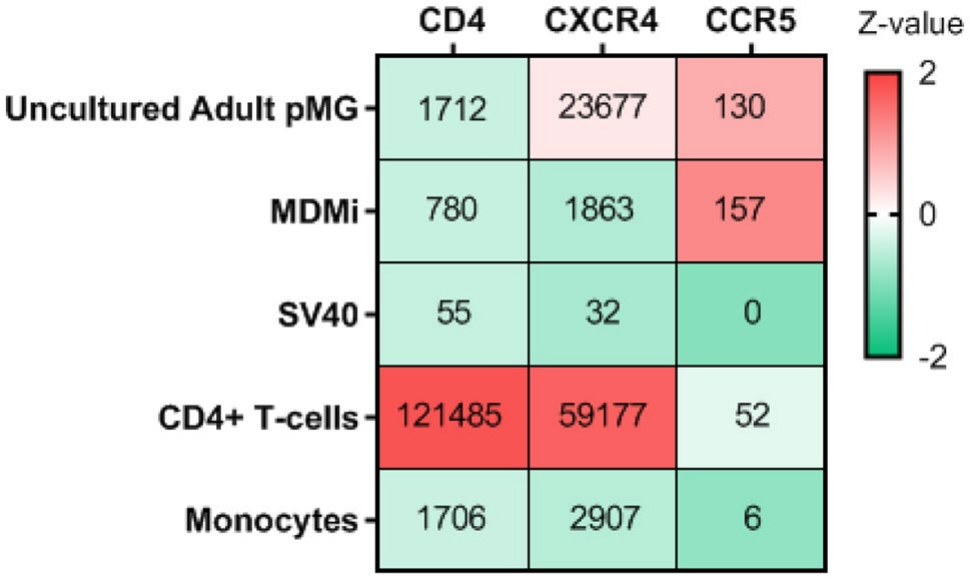
Gene expression of major HIV receptors in in vitro culture models. Median (IQR) gene expression of *CD4*, *CXCR4*, and *CCR5* in primary microglia (pMG), monocyte-derived microglia (MDMi), SV40 microglial cell line, CD4 + T-cells and monocytes assessed by RT-PCR and normalized to the reference gene *GAPDH*. All cells are color-coded according to their *Z*-value (color bar on the right-hand side)

Hereafter, we performed real-time PCR for the main HIV receptor genes, *CD4*, *CXCR4*, and *CCR5,* in three microglial culture models (Fig. 6). *CD4* and *CXCR4* expression were the highest in uncultured adult pMG, followed by MDMi and then SV40 with very low expression. The expression of *CCR5* was slightly higher in MDMi compared to that in uncultured adult pMG and was not expressed in SV40. Interestingly, our real-time PCR data revealed that on a transcriptomic level, uncultured adult pMG express *CXCR4* at considerably higher levels than *CCR5.* This being said, uncultured adult pMG have a substantially lower expression of *CD4* and *CXCR4* and a higher expression of *CCR5* compared to CD4 + T-cells. This further corroborates the theory that R5 M-tropic HIV strains, unlike R5 T-tropic strains, can infect cells expressing relatively low levels of *CD4* including pMG.

## Discussion

Microglia are thought to constitute the main viral reservoir for HIV in the CNS (Wallet et al. 2019), but insight into the dynamics of HIV-microglial interactions has been limited due to the difficulty of obtaining human brain tissues and the limited number of viable microglial cells after isolation. This has led to a boost in the development of novel human microglial culture models, each having its own set of advantages and limitations (Table 4).

**Table 4:** Benefits and limitation of microglial in vitro culture models

Culture model	Benefits	Limitations
Cultured primary microglia	Moderately easy to culture Susceptible to HIV infection	Difficult to obtain fresh human brain tissue Limited number of viable cells Limited life span Transcriptomic deficiencies induced by in vitro culture
Microglial cell lines	Commercially available Easy to culture Mass production Long-term culture Genetic modifications: HIV latency	Transcriptomic profile does not cluster with adult or fetal primary microglia Not susceptible to HIV infection
Monocyte-derived microglia	Easy to obtain and culture Mass production Susceptible to HIV infection	Limited life span Transcriptomic profile does not cluster with adult or fetal primary microglia Expensive
iPSC-derived microglia	Mass production Long-term culture Genetic modifications Susceptible to HIV infection	Transcriptomic profile clusters more closely with fetal microglia Technically complex and time consuming Very expensive
3D organoids	Recapitulate in vivo CNS structure Cell–cell interaction with other CNS cell types Microglia developed in a 3D microenvironment Transcriptomic profile cluster with adult primary microglia Long-term culture	High inter- and intra-variability between organoids Variability in differentiation protocols; patterned and non-patterned Technically complex and time consuming Lack vasculature Ethical concerns Very expensive

The suitability of these models for HIV research, however, remains to be established. We, therefore, aimed to provide researchers in the field of HIV with an encompassing guide to selecting a suitable human microglial in vitro culture model for studying the interplay between HIV and microglia. A good microglial model should strongly resemble uncultured ex vivo adult microglia based on morphology, immune functions, and gene expression profile. Furthermore, the ideal microglial model for HIV research should express HIV receptors and restriction factors at similar levels as uncultured ex vivo adult microglia and support productive HIV infection representative of HIV infection in the CNS. To evaluate the various microglial culture models, we (i) performed a literature review on the morphology, immune functions, expression of microglia-enriched genes, and the susceptibility of each model to HIV infection and (ii) leveraged real-time PCR and RNA-seq data to further characterize the similarity of the various culture models to cultured and uncultured pMG on a transcriptomic level. Our approach, therefore, was novel in so far as to interrogate the transcriptomic data of seven cell types stemming from twelve distinct datasets on a variety of gene signatures to give viability to our interpretation.

The morphology of microglial in vitro culture models is commonly described as having the typical “ramified” morphology of microglia. Although iPSC-MG and oMG showed a closer resemblance to microglia in situ with primary and secondary branches, this is only a minimal representation of the complexity in the arborizations observed in in situ microglia in human brain tissue. Functional characterization of the models based on phagocytosis and LPS responsiveness revealed that every model has the capacity to phagocytose and the ability to induce a pro-inflammatory cytokine response (IL-1β, TNF-α, IL-6). However, most studies did not perform comparative analysis with cultured pMG, rendering it difficult to accurately determine whether these responses are representative. Furthermore, as previously highlighted, these immune functions are not exclusive to microglia. Therefore, we strongly advise to follow up with gene expression profiling, as we did, to more accurately validate the microglial phenotype of a (novel) culture model.

Transcriptome analysis of the microglial models, except for the immortalized cell lines, showed that overall cultured pMG and oMG have the highest level of similarity to the microglial transcriptome gene profile of uncultured adult pMG, followed by iPSC-MG and then MDMi. Focusing on the microglia-specific core gene signature, we noted that none of the models including cultured adult and fetal pMG clustered with uncultured adult pMG. This highlights that unfortunately none of the current microglial models is capable of fully recapitulating the microglial transcriptome of ex vivo pMG analyzed immediately after isolation. Notably, considering the degree of phenotypic changes observed in pMG after culture (Gosselin et al. 2017; Gosselin et al. 2017), it is highly probable that the phenotype of freshly isolated ex vivo pMG does not fully recapitulate the phenotype of in vivo microglia. The most probable cause for this transcriptomic discrepancy is the absence of certain chemical or physical factors from the complex CNS microenvironment and lack of intercellular communication with other CNS cell types (Bohlen et al. 2017; Butovsky et al. 2014). As many of these homeostatic signature genes are involved in the communication between microglia, neurons, and astrocytes, conclusions on the impact of HIV-infected microglia on neurons and neuronal tissue, including the levels of neurotoxicity, should be interpreted with care.

Recent studies revealed that the morphological and transcriptomic deficits can be corrected in part via co-culture with neurons and/or astrocytes (Haenseler et al. 2017; Park et al. 2018; Ryan et al. 2020; Takata et al. 2017), incorporation into 3D cerebral organoids (Abreu et al. 2018; Abud et al. 2017; Brownjohn et al. 2018; dos Reis et al. 2020; Lin et al. 2018; Muffat et al. 2016), or transplantation of iPSC-derived hematopoietic progenitors into humanized mice (Abud et al. 2017; Hasselmann et al. 2019; Mancuso et al. 2019; McQuade et al. 2018; Svoboda et al. 2019). Transplanted microglia acquired a highly ramified morphology, reminiscent of the complex arborization patterns seen in situ, and a gene expression profile that more closely resembled uncultured pMG rather than cultured pMG, including significantly higher expression of key microglia-specific genes such as *P2RY12*, *CX3CR1*, and *CSF1R* (Hasselmann et al. 2019; Mancuso et al. 2019; Svoboda et al. 2019). This indicates that the regulation of key microglial specific genes is dynamically dependent on the environment and can be rescued by mimicking the CNS microenvironment. In this regard, another attempt to drive these cells closer to a microglia fate has been by optimizing the culture media composition with the addition of cytokines (TGF-β, CX3CL1, and CD200) critical for maintaining microglial homeostasis (Abud et al. 2017; Butovsky et al. 2014; Cardona et al. 2006; Hoek et al. 2000; Kierdorf and Prinz 2013) or the use of neural progenitor cell conditioned medium (Banerjee et al. 2020).

Despite the difficulty in maintaining the homeostatic microglial transcriptome in culture, this progressive field of microglial culture models holds great promise for the advancement of HIV research on microglia and the CNS. Thus, to identify which microglial models are suitable for HIV research, we thoroughly investigated the transcriptome similarity of the microglial models, except for cell lines, on the expression of HIV genes that are relevant for HIV infection and replication. HIV receptor expression levels in uncultured adult pMG shared the most similarities with oMG followed by cultured adult pMG. We noted dissimilarities in *CD4* gene expression between cultured and uncultured pMG and a surprisingly low expression of *CD4* and *CXCR4* in iPSC-MG compared to uncultured pMG. A recent study by Rai and colleagues also reported a low expression of both co-receptors in iPSC-MG compared to cultured pMG (Rai et al. 2020). This being said, the expression levels in cultured adult pMG and iPSC-MG differed between datasets corroborating the significance of the culture environment on the microglial transcriptome. The expression of the HIV restriction factors *TRIM5, APOBEC3G*, *and SAMHD1* was generally similar to that in uncultured adult pMG and are mostly conserved between the microglial culture models.

Based on the transcriptomic similarity to uncultured adult pMG across all gene signatures we evaluated, oMG and cultured adult pMG are the most representative culture models when considering HIV research in microglia. However, we acknowledge that cerebral organoids have high inter-organoid variability and that a definitive conclusion should only be drawn upon a transcriptomic evaluation of oMG encompassing a sample size that accounts for such high variability. Furthermore, the statistical approach can be strengthened by leveraging count statistics other than CPM (counts per million) to make the analyses less biased to technical factors evoking artificial variance in the data unassociated with true biological differences between the model systems. Correction approaches accounting for known and unknown technical factors could then be applied without overwriting the interesting biological information within the data.

Infection of the microglial culture models, except for iPSC-MG, with HIV_bal_ exposed distinct differences in virus production. Cultured adult pMG and MDMi both continuously produced virus up to the last day in culture (days 15 and 13). This pattern is consistent with previous studies that showed continuous virus production in cultured pMG and MDMi up to the third week after infection with HIV_bal_ (Albright et al. 2000; Ghorpade et al. 1998; Leone et al. 2006; Rai et al. 2020; Rawat and Spector 2017; Watkins et al. 1990). Interestingly, HIV_bal_ infection of oMG was significantly low compared to that of cultured adult pMG and, contrary to pMG and MDMi, peaked at day 9 and decayed over the following week. On a transcriptomic level, oMG has the closest resemblance to microglia in situ, suggesting that HIV infection in oMG is also the most representative of HIV infection in the CNS. HIV DNA is found irrespective of ART treatment and/or HAND, in a focally distributed small population of CD68 + microglia/macrophage cells (1 to 10%) (Ko et al. 2019; Thompson et al. 2011; Trillo-Pazos et al. 2003; Tso et al. 2018). Besides the sporadic detection of HIV RNA, this indicates that HIV infection, replication, and spread within the microglia population in the CNS is limited. In this regard, the low initial HIV_bal_ infection in oMG is reflective of the small HIV-infected microglial population observed in situ. Interestingly, this decline in viral production after the 1st week of infection was also recently reported by Rai et al. after infection of iPSC-MG with HIV_bal_ (Rai et al. 2020). However, despite being widely used in the HIV research field as an R5 M-tropic virus, HIV_bal_ is a laboratory-adapted strain with replication kinetics and biological properties that might not be representative of R5 M-tropic HIV strains circulating in the human CNS. It will be interesting in future studies to investigate whether this decline in virus production in oMG and iPSC-MG is also observed with viral strains from the CNS of HIV-infected individuals and whether this is due to reversion to a latent state as is proposed for the HIV-infected microglial population in the CNS (Wallet et al. 2019). In summary, different conclusions may be drawn on the level and kinetics of microglia infection, the underlying mechanisms, and potential therapies, based on the model that is chosen. We therefore, propose to be mindful of these potential model-specific effects, and to cross-validate important findings with different models.

Altogether, based on the transcriptome and infection analysis, we find oMG, cultured adult pMG, and iPSC-MG to be suitable microglia in vitro culture models to further research on the interplay between HIV and microglia. However, we acknowledge that all three models, particularly oMG and iPSC-MG, have laborious, costly, and lengthy protocols limiting their widespread use. We do not recommend using the human microglial cell line SV40 or the HMC3-based cell lines for microglia or HIV research as they have large transcriptomic and phenotypic discrepancies with primary microglia and do not support HIV infection. A more affordable, fast, and straightforward model would be MDMi, which is superior to microglial cell lines on a morphological, transcriptional, and functional level. Researchers interested in a cost-effective model with low inter-assay variability for the initial assessment of large cohort studies would benefit from the use of MDMi as a first-line screening tool. Alternatively, we acknowledge the novel hµglia/HIV latent microglial cell line as an exception and a promising model that can be used for the initial assessment of HIV latency reversal on microglia or other HIV latency–related research (Garcia-Mesa et al. 2017). This being said, oMG, cultured pMG, iPSC-MG, and MDMi provide an assessment of HIV-microglia interactions outside of the context of the CNS microenvironment and other CNS cell types. A recent study by Alvarez-Carbonell et al. showed HIV expression in primary microglia was silenced following co-culture with primary neurons (Alvarez-Carbonell et al. 2019). Another study by Ryan et al. showed a reduction in viral production after culturing HIV-infected iPSC-MG with IPSC-derived neurons and astrocytes (Ryan et al. 2020). Thus, it is important to validate experiments done in monoculture in more complex and representative models such as pMG and iPSC-derived co- and tri-culture models or cerebral organoids.

Ultimately, the best model has to be chosen on a case-by-case basis based on the research question and genes of interest and should take into account the capability and resources of the laboratory. We will with no doubt continue to see great technological advancements in this field leading to great improvements in these protocols.

## Methods

### Literature search strategy

For this article, we evaluated five human in vitro microglial culture models and their application in HIV research. We searched the PubMed database for articles describing the generation and characterization of each microglial culture model, including the morphology, inflammatory response, and phagocytic ability of the model. Next, we searched for articles that examined the presence of the HIV genome in microglia in human brain tissue. Finally, we searched for HIV studies performed with human HIV lab strains on any of these culture models.

### Human microglia isolation and culture

Fresh post-mortem brain tissue from the subventricular zone (*n* = 5) was provided by the Netherlands Brain Bank (www.hersenbank.nl). Informed consent was obtained from all donors. Human microglia were isolated and cultured according to the protocol described before (Sneeboer et al. 2019). In short, a mechanical and enzymatic dissociation with DNAse 1 (200 µg/ml; Roche Diagnostics GmbH) and trypsin was done to obtain a single cell suspension, followed by a Percoll gradient to remove myelin and red blood cells. Microglia enrichment was achieved by positive selection for CD11b expression using CD11b + MACS (Miltenyi Biotec, Germany) according to the manufacturer’s protocol. Microglial cells were cultured in 200 μl microglia medium ((RPMI 1640; Gibco Life Technologies, USA) supplemented with 10% FCS, 1% penicillin–streptomycin (Gibco Life Technologies, USA) and 100 ng/ml IL-34 (Miltenyi Biotec, Germany)) for 1 day before infection.

### Isolation and culture of other microglial cell models

The SV40 human immortalized microglial cell line, originally derived from microglia isolated from the embryonic spinal cord and cortex immortalized with SV40 virus, was obtained from Applied Biological Materials Inc. (Janabi et al. 1995). SV40 were maintained in RPMI 1640 (Gibco Life Technologies, USA) supplemented with 10% FCS and 1% penicillin–streptomycin (Gibco Life Technologies, USA).

Monocytes were isolated from PBMCs by CD14 + MACS (Miltenyi Biotec, Germany) according to the manufacturer’s protocol. Monocytes were then differentiated to monocyte-derived microglia according to the protocol of (Ormel et al. 2020). In short, monocytes were cultured in monocyte culture medium (RPMI 1640 (Gibco Life Technologies, USA), 2 mM l-glutamine, 1% penicillin–streptomycin (Gibco Life Technologies, USA)) + 25% astrocyte-conditioned medium (ACM) (SCC1811, ScienCell, USA). On the fourth and eighth day in culture, the medium was replaced with monocyte-derived microglia (MDMi) medium (RPMI 1640, 2 mM l-glutamine, 1% penicillin–streptomycin, 25% ACM, 10 ng/ml M-CSF, 10 ng/ml GM-CSF, 20 ng/ ml TGFβ, 12.5 ng/ml IFNγ, and 100 ng/ml IL34 (all cytokines from Miltenyi Biotec, Germany)). Infection and qPCR analyses were done on day 10 post differentiation.

Three-dimensional cerebral organoids were generated as we have published before (Ormel et al. 2018). Three-dimensional organoids were dissociated with enzymatic dissociation using papain (18.6 U/ml, Worthington, LK003176) and DNAse 1 (337 U/ml Worthington, LK003170), followed by microglia isolation using CD11b + MACS (Miltenyi Biotec, Germany) according to the protocol published before (Ormel et al. 2018). Organoid microglia (oMG) were cultured in poly-l-lysine hydrobromide (PLL)–coated 96-well plates in microglia medium. Infection experiments were performed on day 1 post isolation.

### Construction of HIV-1 reporter virus

An HXB2 molecular clone (pHXB2PS) was used to construct a molecular gp160 deletion vector with a luciferase reporter gene (HxB2ΔENVluc). pHXB2PS is derived from pHXB2WT (Van Maarseveen et al. 2006), which expresses the full-length HIV-1 sequence HXB2 (9719 bp, GenBank accession number K03455.1), with all bacterial sequences non-essential for bacterial expression and replication removed. To create the HxB2 env deletion vector, a unique BtgZI site was introduced at position 6112 in pHxB2PS by site-directed mutagenesis PCR. The envelope coding region was removed through digestion with BtgZI and BsmBI (6112–8850) and replaced with a linker sequence. Hereafter, we cloned the NanoLuc luciferase gene (pNL1.3) (Promega) into the nef gene using the unique restriction site Bpu1102I as described before (Lebbink et al. 2017). Undesired NgoMIV and BtgZI restriction sites in the NanoLuc luciferase gene were removed by silent mutation to facilitate envelope cloning.

To generate the HxB2bal luciferase reporter virus (HxB2Balgp160Luc), we first amplified the envelop coding region of the R5 laboratory-adapted HIV-1 strain BaL (obtained through the NIH HIV Reagent Programs (https://www.hivreagentprogram.org/)), using the SuperScript III one-step RT-PCR system with Platinum *Taq* High Fidelity DNA Polymerase (Thermo Fisher Scientific, USA), according to the manufacturer’s protocol. The real-time RT-PCR reaction was done with the primers Oevif-1forw 5′-GGTCAGGGAGTCTCCATAGAATGGAGG-3′ and HIV-R-end-rev1 5′-GCACACAACGCGTGAAGCACTCAAGGCAAGCTTTATTGAGGC-3′, followed by a nested PCR with the primers gp160-fw 5′-TAGTAGTAGCASYAATCATCGCAATAGTTGTGTGG-3′ and gp160-rv 5′-CTCGTCTCATTCTTTCCCTTACMKCAGGCCATCC-3′. The PCR product and the HxB2ΔENVluc vector were digested with BtgZI (6112) and BsmBI (8850) and subsequently ligated with T4 ligase.

To generate the NL4-3bal GFP reporter virus (NL4-3Balgp160GFP), we cloned the envelope coding region of HIV-1 BaL into an NL4-3 GFP reporter molecular clone (NL4-3GFPwt) (a kind gift from Theo Geijtenbeek (AMC, the Netherlands)) using the unique restriction sites SalI (5785) and NotI (8797). In brief, two gBlock gene fragments (Integrated DNA Technologies (IDT)) encoding for the HIV-1 BaL envelope gene and a 25-nt overlap with the NL4-3GFPwt vector at both the 5′ and 3′ ends were cloned into the NL4-3GFPwt molecular clone using the NEBuilder® HiFi DNA Assembly Master Mix kit following the manufacturer’s instructions (New England BioLabs). We also introduced two silent mutations in the vpu gene to create a new, unique AfeI restriction site at position 6091/6092 to facilitate envelope cloning. Single colonies were picked and expanded, and plasmid DNA was isolated using the GeneJET Plasmid Miniprep Kit (Thermo Scientific). All HIV constructs were verified by nucleotide sequencing.

Replication competent viral stocks were generated by transfecting HEK-293 T cells with the chimeric infectious plasmids (HxB2Balgp160Luc and NL4-3Balgp160GFP) using Lipofectamine 2000 reagent (Invitrogen). The supernatant was harvested at 48 h.

### Infection of microglia culture models

Cultured adult pMG, MDMi, oMG, and SV40 cells (1 × 10^5^) were infected with 10 ng (p24 Gag) of HIVbalLuc (HXB2Balgp160Luc) or HIVbalGFP (NL4-3Balgp160GFP). The virus was washed away the next day, and cells were cultured in their respective culture medium for 13–15 days without medium refreshment. The supernatant was collected 2–3×/week. Luminescence was measured using the Nano-Glo® Luciferase Assay System (Promega) according to the manufacturer’s protocol. Experiments were carried out in duplicate or triplicate. Graphs were generated with GraphPad Prism version 8.3.0 (GraphPad Software) and depict the mean and range.

### RNA isolation and quantitative PCR

RNA was isolated from adult primary microglia, MDMi, SV40, CD4 + T-cells, and monocytes using the RNeasy kit (Qiagen, the Netherlands) including the DNAse treatment according to the manufacturer’s protocol. RNA isolation and downstream gene expression analysis were done in duplicate from 4 (pMG, monocytes) or 5 (CD4 + T cells, MDMi) different donors, except for SV40. cDNA synthesis was performed with the iScript cDNA synthesis kit (Bio-Rad) according to the manufacturer’s protocol. qPCR was done in a 7900 Real Time PCR System (Applied Biosystems) with the following cycle conditions: 95 °C for 10 min, 40 cycles at 95 °C of 15 s, and 60 °C for 60 s. Per reaction, 5 µl SYBR green PCR Master Mix (Roche; Life Technologies Corporation, Grand Island, NY), 1 µl primer mix (2 pmol/ml), and 5 ng RNA were added up to a final volume of 10 µl. Primer sequences are listed in Supplementary Table 1. Transcript levels of glyceraldehyde 3-phosphate dehydrogenase (GAPDH) appeared to be most stable and were used for normalization. Quantification was done by raising 2 to the power of the negative CT values, and absolute expression was then calculated by dividing the CT values of the samples with GAPDH and then multiplying by 10.000. Median, interquartile range, and standard deviation were calculated for each gene using GraphPad Prism version 8.3.0 (GraphPad Software). *Z*-values were calculated for each gene by subtracting the mean and then dividing by the standard deviation. A heatmap was generated using GraphPad Prism version 8.3.0 (GraphPad Software).

### RNA sequencing

The transcriptomic phenotype can be efficiently profiled by sequencing the RNA content of bulk tissues containing up to millions of cells (i.e., bulk RNA-seq; Gawad et al. 2016; Stegle et al. 2015). Cell-type specificity is arguably a criticism when sequencing heterogeneous bulk tissues that contain a multiplicity of cell types but can be achieved by focusing sequencing capacities on one specific cell type. To evaluate transcriptomic similarity across microglia models, we, therefore, selected published microglia model system-specific RNA-seq datasets of adult pMG (Gosselin et al. 2017; Ormel et al. 2018, 2020), cultured pMG (Gosselin et al. 2017; Lopes et al. 2020; Ormel et al. 2018), fetal pMG (Douvaras et al. 2017), monocytes (Gosselin et al. 2017; Ormel et al. 2020), MDMi (Ormel et al. 2020), iPSC-derived microglia (Brownjohn et al. 2018; Douvaras et al. 2017), and organoid-derived microglia (Ormel et al. 2018); integrated these data into one dataset; and subsequently analyzed it in R v4.0.3. We selected these datasets in June 2020.

### Data pre-processing

Where raw counts were available, genes with less than one count per sample were removed from the analysis. Then, counts were normalized to log counts per million (logCPM) using the cpm command from edgeR v3.14.0 (Robinson et al. 2009) with setting prior counts to 1 and taking the logarithmic. Subsequently, the normalized datasets were merged. Only healthy and unstimulated samples were included. The final dataset consisted of 129 samples from 12 datasets containing 7 distinct microglia model systems. Sample and gene outlier detection was performed using interquartile range measures on Pearson correlation with outliers being defined as 3 standard deviations above or below the mean. No samples or genes were detected as outliers. To account for technical bias leading to dataset differences, surrogate variable– and principal component–based correction approaches were applied using the sva v3.20.0 (Leek et al. 2012) and limma v3.28.14 (Smyth 2005) packages. Post hoc evaluations of the correction approaches by leveraging k-means clustering, unsupervised hierarchical clustering, and PCA, however, showed no sample clustering based on cell-type identity. Instead, no data correction was performed as the validity of this approach was supported through inter-dataset similarity of monocyte and ex vivo primary microglia samples as indicated on PCA and unsupervised hierarchical clustering (see results). Plots were generated using the ggplot2 v3.3.2 package.

### PCA, k-means clustering, and unsupervised hierarchical clustering

Pearson correlation was performed using the default rcorr function from the Hmisc v4.4.1 package. PCA was executed on samples using the prcomp function, scaling and centering the data prior. k-means clustering of samples was performed with the kmeans function using the Hartigan–Wong algorithm. The optimum number of clusters was calculated using the fviz_nbclust function from the factoextra v1.0.7 package. Hierarchical clustering was performed with the pheatmap v1.0.12 package using Euclidean distances.

## Supplementary information

Below is the link to the electronic supplementary material.

Supplementary file1 (PDF 591 kb)

## References

[CR1] Abe N, Nishihara T, Yorozuya T, Tanaka J (2020). Microglia and macrophages in the pathological central and peripheral nervous systems. Cells.

[CR2] Abreu CM, Gama L, Krasemann S, Chesnut M, Odwin-Dacosta S, Hogberg HT, Hartung T, Pamies D (2018). Microglia increase inflammatory responses in iPSC-derived human BrainSpheres. Front Microbiol.

[CR3] Abud EM, Ramirez RN, Martinez ES, Healy LM, Nguyen CHH, Newman SA, Yeromin AV, Scarfone VM, Marsh SE, Fimbres C, Caraway CA, Fote GM, Madany AM, Agrawal A, Kayed R, Gylys KH, Cahalan MD, Cummings BJ, Antel JP, Mortazavi A, Carson MJ, Poon WW, Blurton-Jones M (2017). iPSC-derived human microglia-like cells to study neurological diseases. Neuron.

[CR4] Akhter R, Shao Y, Formica S, Khrestian M, Bekris LM (2021). TREM2 alters the phagocytic, apoptotic and inflammatory response to Aβ42 in HMC3 cells. Mol Immunol.

[CR5] Albright AV, Shieh JTC, Itoh T, Lee B, Pleasure D, O’Connor MJ, Doms RW, González-Scarano F (1999). Microglia express CCR5, CXCR4, and CCR3, but of these, CCR5 is the principal coreceptor for human immunodeficiency virus type 1 dementia isolates. J Virol.

[CR6] Albright AV, Shieh JTC, O’Connor MJ, González-Scarano F (2000). Characterization of cultured microglia that can be infected by HIV-1. J Neurovirol.

[CR7] Alvarez-Carbonell D, Garcia-Mesa Y, Milne S, Das B, Dobrowolski C, Rojas R, Karn J (2017). Toll-like receptor 3 activation selectively reverses HIV latency in microglial cells. Retrovirology.

[CR8] Alvarez-Carbonell D, Ye F, Ramanath N, Garcia-Mesa Y, Knapp PE, Hauser KF, Karn J (2019). Cross-talk between microglia and neurons regulates HIV latency. PLoS Pathog.

[CR9] Ambrosius B, Faissner S, Guse K, von Lehe M, Grunwald T, Gold R, Grewe B, Chan A (2017). Teriflunomide and monomethylfumarate target HIV-induced neuroinflammation and neurotoxicity. J Neuroinflammation.

[CR10] Amos PJ, Fung S, Case A, Kifelew J, Osnis L, Smith CL, Green K, Naydenov A, Aloi M, Hubbard JJ, Ramakrishnan A, Garden GA, Jayadev S (2017). Modulation of hematopoietic lineage specification impacts TREM2 expression in microglia-like cells derived from human stem cells. ASN Neuro.

[CR11] Ances BM, Anderson AM, Letendre SL (2021). CROI 2021: Neurologic complications of HIV-1 infection or COVID-19. Top Antivir Med.

[CR12] Ances BM, Letendre SL (2019). CROI 2019: neurologic complications of HIV disease. Top Antivir Med.

[CR13] Ancuta P, Kunstman KJ, Autissier P, Zaman T, Stone D, Wolinsky SM, Gabuzda D (2006). CD16+ monocytes exposed to HIV promote highly efficient viral replication upon differentiation into macrophages and interaction with T cells. Virology.

[CR14] Arrildt KT, LaBranche CC, Joseph SB, Dukhovlinova EN, Graham WD, Ping L-H, Schnell G, Sturdevant CB, Kincer LP, Mallewa M, Heyderman RS, Van Rie A, Cohen MS, Spudich S, Price RW, Montefiori DC, Swanstrom R (2015). Phenotypic correlates of HIV-1 macrophage tropism. J Virol.

[CR15] Asahchop EL, Meziane O, Mamik MK, Chan WF, Branton WG, Resch L, Gill MJ, Haddad E, Guimond JV, Wainberg MA, Baker GB, Cohen EA, Power C (2017). Reduced antiretroviral drug efficacy and concentration in HIV-infected microglia contributes to viral persistence in brain. Retrovirology.

[CR16] Banerjee P, Paza E, Perkins EM, James OG, Kenkhuis B, Lloyd AF, Burr K, Story D, Yusuf D, He X, Backofen R, Dando O, Chandran S, Priller J (2020). Generation of pure monocultures of human microglia-like cells from induced pluripotent stem cells. Stem Cell Res.

[CR17] Bednar MM, Sturdevant CB, Tompkins LA, Arrildt KT, Dukhovlinova E, Kincer LP, Swanstrom R (2015). Compartmentalization, viral evolution, and viral latency of HIV in the CNS. Curr HIV/AIDS Rep.

[CR18] Bennett ML, Bennett FC, Liddelow SA, Ajami B, Zamanian JL, Fernhoff NB, Mulinyawe SB, Bohlen CJ, Adil A, Tucker A, Weissman IL, Chang EF, Li G, Grant GA, Hayden Gephart MG, Barres BA (2016). New tools for studying microglia in the mouse and human CNS. Proc Natl Acad Sci.

[CR19] Bertin J, Barat C, Bélanger D, Tremblay MJ (2012). Leukotrienes inhibit early stages of HIV-1 infection in monocyte-derived microglia-like cells. J Neuroinflammation.

[CR20] Bohlen CJ, Bennett FC, Tucker AF, Collins HY, Mulinyawe SB, Barres BA (2017). Diverse requirements for microglial survival, specification, and function revealed by defined-medium cultures. Neuron.

[CR21] Böttcher C, Schlickeiser S, Sneeboer MAM, Kunkel D, Knop A, Paza E, Fidzinski P, Kraus L, Snijders GJL, Kahn RS, Schulz AR, Mei HE, Hol EM, Siegmund B, Glauben R, Spruth EJ, de Witte LD, Priller J (2019). Human microglia regional heterogeneity and phenotypes determined by multiplexed single-cell mass cytometry. Nat Neurosci.

[CR22] Brese RL, Gonzalez-Perez MP, Koch M, O’Connell O, Luzuriaga K, Somasundaran M, Clapham PR, Dollar JJ, Nolan DJ, Rose R, Lamers SL (2018). Ultradeep single-molecule real-time sequencing of HIV envelope reveals complete compartmentalization of highly macrophage-tropic R5 proviral variants in brain and CXCR4-using variants in immune and peripheral tissues. J Neurovirol.

[CR23] Brownjohn PW, Smith J, Solanki R, Lohmann E, Houlden H, Hardy J, Dietmann S, Livesey FJ (2018). Functional studies of missense TREM2 mutations in human stem cell-derived microglia. Stem Cell Reports.

[CR24] Budka H, Costanzi G, Cristina S, Lechi A, Parravicini C, Trabattoni R, Vago L (1987). Brain pathology induced by infection with the human immunodeficiency virus (HIV) - a histological, immunocytochemical, and electron microscopical study of 100 autopsy cases. Acta Neuropathol.

[CR25] Butovsky O, Jedrychowski MP, Moore CS, Cialic R, Lanser AJ, Gabriely G, Koeglsperger T, Dake B, Wu PM, Doykan CE, Fanek Z, Liu L, Chen Z, Rothstein JD, Ransohoff RM, Gygi SP, Antel JP, Weiner HL (2014). Identification of a unique TGF-β-dependent molecular and functional signature in microglia. Nat Neurosci.

[CR26] Calcagno A, Di Perri G, Bonora S (2017). Treating HIV infection in the central nervous system. Drugs.

[CR27] Campbell LA, Coke LM, Richie CT, Fortuno LV, Park AY, Harvey BK (2019). Gesicle-mediated delivery of CRISPR/Cas9 ribonucleoprotein complex for inactivating the HIV provirus. Mol Ther.

[CR28] Campbell LA, Richie CT, Zhang Y, Heathward EJ, Coke LM, Park EY, Harvey BK (2017). In vitro modeling of HIV proviral activity in microglia. FEBS J.

[CR29] Cardona AE, Pioro EP, Sasse ME, Kostenko V, Cardona SM, Dijkstra IM, Huang DR, Kidd G, Dombrowski S, Dutta R, Lee JC, Cook DN, Jung S, Lira SA, Littman DR, Ransohoff RM (2006). Control of microglial neurotoxicity by the fractalkine receptor. Nat Neurosci.

[CR30] Caruana G, Vidili G, Serra PA, Bagella P, Spanu A, Fiore V, Calvisi DF, Manetti R, Rocchitta G, Nuvoli S, Babudieri S, Simile MM, Madeddu G (2017). The burden of HIV-associated neurocognitive disorder (HAND) in post-HAART era: a multidisciplinary review of the literature. Eur Rev Med Pharmacol Sci.

[CR31] Castellano P, Prevedel L, Eugenin EA (2017). HIV-infected macrophages and microglia that survive acute infection become viral reservoirs by a mechanism involving Bim. Sci Rep.

[CR32] Cenker JJ, Stultz RD, McDonald D (2017). Brain microglial cells are highly susceptible to HIV-1 infection and spread. AIDS Res Hum Retroviruses.

[CR33] Chai Q, Jovasevic V, Malikov V, Sabo Y, Morham S, Walsh D, Naghavi MH (2017). HIV-1 counteracts an innate restriction by amyloid precursor protein resulting in neurodegeneration. Nat Commun.

[CR34] Chan WK, Fetit R, Griffiths R, Marshall H, Mason JO, Price DJ (2021). Using organoids to study human brain development and evolution. Dev Neurobiol.

[CR35] Cherrier T, Elias M, Jeudy A, Gotthard G, Le Douce V, Hallay H, Masson P, Janossy A, Candolfi E, Rohr O, Chabrière E, Schwartz C (2011). Human-phosphate-binding-protein inhibits HIV-1 gene transcription and replication. Virol J.

[CR36] Chiaradia I, Lancaster MA (2020). Brain organoids for the study of human neurobiology at the interface of in vitro and in vivo. Nat Neurosci.

[CR37] Chiavari M, Ciotti GMP, Navarra P, Lisi L (2019). Pro-inflammatory activation of a new immortalized human microglia cell line. Brain Sci.

[CR38] Chitu V, Gokhan Ş, Nandi S, Mehler MF, Stanley ER (2016). Emerging roles for CSF-1 receptor and its ligands in the nervous system. Trends Neurosci.

[CR39] Chugh P, Fan S, Planelles V, Maggirwar SB, Dewhurst S, Kim B (2007). Infection of human immunodeficiency virus and intracellular viral tat protein exert a pro-survival effect in a human microglial cell line. J Mol Biol.

[CR40] Churchill MJ, Gorry PR, Cowley D, Lal L, Sonza S, Purcell DF, Thompson KA, Gabuzda D, McArthur JC, Pardo CA, Wesselingh SL (2006). Use of laser capture microdissection to detect integrated HIV-1 DNA in macrophages and astrocytes from autopsy brain tissues. J Neurovirol.

[CR41] Ciborowski P, Kadiu I, Rozek W, Smith L, Bernhardt K, Fladseth M, Ricardo-Dukelow M, Gendelman HE (2007). Investigating the human immunodeficiency virus type 1-infected monocyte-derived macrophage secretome. Virology.

[CR42] Colonna M (2003). Trems in the immune system and beyond. Nat Rev Immunol.

[CR43] Colonna M, Butovsky O (2017). Microglia function in the central nervous system during health and neurodegeneration. Annu Rev Immunol.

[CR44] Davalos D, Grutzendler J, Yang G, Kim JV, Zuo Y, Jung S, Littman DR, Dustin ML, Gan WB (2005). ATP mediates rapid microglial response to local brain injury in vivo. Nat Neurosci.

[CR45] Davies DE, Lloyd JB (1989). Monocyte-to-macrophage transition in vitro. A systematic study using human cells isolated by fractionation on Percoll. J Immunol Methods.

[CR46] Davis LE, Hjelle BL, Miller VE, Palmer DL, Llewellyn AL, Merlin TL, Young SA, Mills RG, Wachsman W, Wiley CA (1992). Early viral brain invasion in iatrogenic human immunodeficiency virus infection. Neurology.

[CR47] Delaney MK, Malikov V, Chai Q, Zhao G, Naghavi MH (2017). Distinct functions of diaphanous-related formins regulate HIV-1 uncoating and transport. Proc Natl Acad Sci U S A.

[CR48] Dello Russo C, Cappoli N, Coletta I, Mezzogori D, Paciello F, Pozzoli G, Navarra P, Battaglia A (2018). The human microglial HMC3 cell line: where do we stand?. A Systematic Literature Review J Neuroinflammation.

[CR49] DePaula-Silva AB, Gorbea C, Doty DJ, Libbey JE, Sanchez JMS, Hanak TJ, Cazalla D, Fujinami RS (2019). Differential transcriptional profiles identify microglial- and macrophage-specific gene markers expressed during virus-induced neuroinflammation. J Neuroinflammation.

[CR50] dos Reis RS, Sant S, Keeney H, Wagner MCE, Ayyavoo V (2020). Modeling HIV-1 neuropathogenesis using three-dimensional human brain organoids (hBORGs) with HIV-1 infected microglia. Sci Rep.

[CR51] Douvaras P, Sun B, Wang M, Kruglikov I, Lallos G, Zimmer M, Terrenoire C, Zhang B, Gandy S, Schadt E, Freytes DO, Noggle S, Fossati V (2017). Directed differentiation of human pluripotent stem cells to microglia. Stem Cell Reports.

[CR52] Enting RH, Prins JM, Jurriaans S, Brinkman K, Portegies P, Lange JMA (2001). Concentrations of human immunodeficiency virus type 1 (HIV-1) RNA in cerebrospinal fluid after antiretroviral treatment inflated during primary HIV-1 infection. Clin Infect Dis.

[CR53] Erblich B, Zhu L, Etgen AM, Dobrenis K, Pollard JW (2011). Absence of colony stimulation factor-1 receptor results in loss of microglia, disrupted brain development and olfactory deficits. PLoS One.

[CR54] Etemad S, Zamin RM, Ruitenberg MJ, Filgueira L (2012). A novel in vitro human microglia model: characterization of human monocyte-derived microglia. J Neurosci Methods.

[CR55] Evans TA, Barkauskas DS, Myers JT, Hare EG, You JQ, Ransohoff RM, Huang AY, Silver J (2014). High-resolution intravital imaging reveals that blood-derived macrophages but not resident microglia facilitate secondary axonal dieback in traumatic spinal cord injury. Exp Neurol.

[CR56] Fairman P, Angel JB (2012). The effect of human immunodeficiency virus-1 on monocyte-derived dendritic cell maturation and function. Clin Exp Immunol.

[CR57] Francis AC, Marin M, Prellberg MJ, Palermino-Rowland K, Melikyan GB (2020). HIV-1 uncoating and nuclear import precede the completion of reverse transcription in cell lines and in primary macrophages. Viruses.

[CR58] Galatro TF, Holtman IR, Lerario AM, Vainchtein ID, Brouwer N, Sola PR, Veras MM, Pereira TF, Leite REP, Möller T, Wes PD, Sogayar MC, Laman JD, Den Dunnen W, Pasqualucci CA, Oba-Shinjo SM, Boddeke EWGM, Marie SKN, Eggen BJL (2017). Transcriptomic analysis of purified human cortical microglia reveals age-associated changes. Nat Neurosci.

[CR59] Galloway DA, Phillips AEM, Owen DRJ, Moore CS (2019). Corrigendum: Phagocytosis in the brain: homeostasis and disease. Front Immunol.

[CR60] Garcia-Mesa Y, Jay TR, Checkley MA, Luttge B, Dobrowolski C, Valadkhan S, Landreth GE, Karn J, Alvarez-Carbonell D (2017). Immortalization of primary microglia: a new platform to study HIV regulation in the central nervous system. J Neurovirol.

[CR61] Gautiar EL, Shay T, Miller J, Greter M, Jakubzick C, Ivanov S, Helft J, Chow A, Elpek KG, Gordonov S, Mazloom AR, MaAyan A, Chua WJ, Hansen TH, Turley SJ, Merad M, Randolph GJ, Best AJ, Knell J, Immunological Genome Consortium (2012). Gene-expression profiles and transcriptional regulatory pathways that underlie the identity and diversity of mouse tissue macrophages. Nat Immunol.

[CR62] Gawad C, Koh W, Quake SR (2016). Single-cell genome sequencing: current state of the science. Nat Rev Genet.

[CR63] Geirsdottir L, David E, Keren-Shaul H, Weiner A, Bohlen SC, Neuber J, Balic A, Giladi A, Sheban F, Dutertre CA, Pfeifle C, Peri F, Raffo-Romero A, Vizioli J, Matiasek K, Scheiwe C, Meckel S, Mätz-Rensing K, van der Meer F, Thormodsson FR, Stadelmann C, Zilkha N, Kimchi T, Ginhoux F, Ulitsky I, Erny D, Amit I, Prinz M (2019). Cross-species single-cell analysis reveals divergence of the primate microglia program. Cell.

[CR64] Ghorpade A, Nukuna A, Che M, Haggerty S, Persidsky Y, Carter E, Carhart L, Shafer L, Gendelman HE (1998). Human immunodeficiency virus neurotropism: an analysis of viral replication and cytopathicity for divergent strains in monocytes and microglia. J Virol.

[CR65] Global HIV & AIDS statistics (2020) fact sheet | UNAIDS. Available from https://www.unaids.org/en/resources/fact-sheet. Accessed 6 MAY 2021

[CR66] Gosselin D, Skola D, Coufal NG, Holtman IR, Schlachetzki JCM, Sajti E, Jaeger BN, O’Connor C, Fitzpatrick C, Pasillas MP, Pena M, Adair A, Gonda DD, Levy ML, Ransohoff RM, Gage FH, Glass CK (2017). An environment-dependent transcriptional network specifies human microglia identity. Science.

[CR67] Greenhalgh AD, David S (2014). Differences in the phagocytic response of microglia and peripheral macrophages after spinal cord Injury and its effects on cell death. J Neurosci.

[CR68] Grenier Y, Ruijs TCG, Robitaille Y, Olivier A, Antel JP (1989). Immunohistochemical studies of adult human glial cells. J Neuroimmunol.

[CR69] Haage V, Semtner M, Vidal RO, Hernandez DP, Pong WW, Chen Z, Hambardzumyan D, Magrini V, Ly A, Walker J, Mardis E, Mertins P, Sauer S, Kettenmann H, Gutmann DH (2019). Comprehensive gene expression meta-analysis identifies signature genes that distinguish microglia from peripheral monocytes/macrophages in health and glioma. Acta Neuropathol Commun.

[CR70] Haenseler W, Rajendran L (2019). Concise review: modeling neurodegenerative diseases with human pluripotent stem cell-derived microglia. Stem Cells.

[CR71] Haenseler W, Sansom SN, Buchrieser J, Newey SE, Moore CS, Nicholls FJ, Chintawar S, Schnell C, Antel JP, Allen ND, Cader MZ, Wade-Martins R, James WS, Cowley SA (2017). A highly efficient human pluripotent stem cell microglia model displays a neuronal-co-culture-specific expression profile and inflammatory response. Stem Cell Reports.

[CR72] Harrington PR, Schnell G, Letendre SL, Ritola K, Robertson K, Hall C, Burch CL, Jabara CB, Moore DT, Ellis RJ, Price RW, Swanstrom R (2009). Cross-sectional characterization of HIV-1 env compartmentalization in cerebrospinal fluid over the full disease course. AIDS.

[CR73] Hassan NF, Campbell DE, Rifat S, Douglas SD (1991). Isolation and characterization of human fetal brain-derived microglia in in vitro culture. Neuroscience.

[CR74] Hasselmann J, Blurton-Jones M (2020). Human iPSC-derived microglia: a growing toolset to study the brain’s innate immune cells. Glia.

[CR75] Hasselmann J, Coburn MA, England W, Figueroa Velez DX, Kiani Shabestari S, Tu CH, McQuade A, Kolahdouzan M, Echeverria K, Claes C, Nakayama T, Azevedo R, Coufal NG, Han CZ, Cummings BJ, Davtyan H, Glass CK, Healy LM, Gandhi SP, Spitale RC, Blurton-Jones M (2019). Development of a chimeric model to study and manipulate human microglia in vivo. Neuron.

[CR76] Hayes GM, Woodroofe MN, Cuzner ML (1988). Characterisation of microglia isolated from adult human and rat brain. J Neuroimmunol.

[CR77] Heaton RK, Clifford DB, Franklin DR, Woods SP, Ake C, Vaida F, Ellis RJ, Letendre SL, Marcotte TD, Atkinson JH, Rivera-Mindt M, Vigil OR, Taylor MJ, Collier AC, Marra CM, Gelman BB, McArthur JC, Morgello S, Simpson DM, McCutchan JA, Abramson I, Gamst A, Fennema-Notestine C, Jernigan TL, Wong J, Grant I (2010). HIV-associated neurocognitive disorders persist in the era of potent antiretroviral therapy: Charter study. Neurology.

[CR78] Heaton RK, Franklin DR, Ellis RJ, McCutchan JA, Letendre SL, LeBlanc S, Corkran SH, Duarte NA, Clifford DB, Woods SP, Collier AC, Marra CM, Morgello S, Rivera Mindt M, Taylor MJ, Marcotte TD, Atkinson JH, Wolfson T, Gelman BB, McArthur JC, Simpson DM, Abramson I, Gamst A, Fennema-Notestine C, Jernigan TL, Wong J, Grant I, CHARTER Group, HNRC Group (2011). HIV-associated neurocognitive disorders before and during the era of combination antiretroviral therapy: differences in rates, nature, and predictors. J Neurovirol.

[CR79] Heaton RK, Franklin DR, Deutsch R, Letendre S, Ellis RJ, Casaletto K, Marquine MJ, Woods SP, Vaida F, Atkinson JH, Marcotte TD, McCutchan JA, Collier AC, Marra CM, Clifford DB, Gelman BB, Sacktor N, Morgello S, Simpson DM, Abramson I, Gamst AC, Fennema-Notestine C, Smith DM, Grant I, CHARTER Group (2015). Neurocognitive change in the era of HIV combination antiretroviral therapy: the Longitudinal CHARTER study. Clin Infect Dis.

[CR80] Hendrickx DAE, Schuurman KG, van Draanen M, Hamann J, Huitinga I (2014). Enhanced uptake of multiple sclerosis-derived myelin by THP-1 macrophages and primary human microglia. J Neuroinflammation.

[CR81] Hickman SE, Kingery ND, Ohsumi TK, Borowsky ML, Wang LC, Means TK, El Khoury J (2013). The microglial sensome revealed by direct RNA sequencing. Nat Neurosci.

[CR82] Hoek RH, Ruuls SR, Murphy CA, Wright GJ, Goddard R, Zurawski SM, Blom B, Homola ME, Streit WJ, Brown MH, Barclay AN, Sedgwick JD (2000). Down-regulation of the macrophage lineage through interaction with OX2 (CD200). Science.

[CR83] Huang Y, Zhao L, Jia B, Wu L, Li Y, Curthoys N, Zheng JC (2011). Glutaminase dysregulation in HIV-1-infected human microglia mediates neurotoxicity: relevant to HIV-1-associated neurocognitive disorders. J Neurosci.

[CR84] Imai Y, Ibata I, Ito D, Ohsawa K, Kohsaka S (1996). A novel gene iba1 in the major histocompatibility complex class III region encoding an EF hand protein expressed in a monocytic lineage. Biochem Biophys Res Commun.

[CR85] Ingram Z, Taylor M, Okland G, Martin R, Hulme AE (2020). Characterization of HIV-1 uncoating in human microglial cell lines. Virol J.

[CR86] Jadhav VS, Krause KH, Singh SK (2014). HIV-1 Tat C modulates NOX2 and NOX4 expressions through miR-17 in a human microglial cell line. J Neurochem.

[CR87] Janabi N, Peudenier S, Héron B, Ng KH, Tardieu M (1995). Establishment of human microglial cell lines after transfection of primary cultures of embryonic microglial cells with the SV40 large T antigen. Neurosci Lett.

[CR88] Jones BA, Beamer M, Ahmed S (2010). Fractalkine/CX3CL1: a potential new target for inflammatory diseases. Mol Interv.

[CR89] Joseph SB, Swanstrom R (2018). The evolution of HIV-1 entry phenotypes as a guide to changing target cells. J Leukoc Biol.

[CR90] Joseph SB, Swanstrom R, Kashuba ADM, Cohen MS (2015). Bottlenecks in HIV-1 transmission: insights from the study of founder viruses. Nat Rev Microbiol.

[CR91] Kierdorf K, Erny D, Goldmann T, Sander V, Schulz C, Perdiguero EG, Wieghofer P, Heinrich A, Riemke P, Hölscher C, Müller DN, Luckow B, Brocker T, Debowski K, Fritz G, Opdenakker G, Diefenbach A, Biber K, Heikenwalder M, Geissmann F, Rosenbauer F, Prinz M (2013). Microglia emerge from erythromyeloid precursors via Pu.1-and Irf8-dependent pathways. Nat Neurosci.

[CR92] Kierdorf K, Prinz M (2013). Factors regulating microglia activation. Front Cell Neurosci.

[CR93] Kierdorf K, Prinz M (2017). Microglia in steady state. J Clin Invest.

[CR94] Kim J, Sullivan GJ, Park IH (2021) How well do brain organoids capture your brain?. iScience 24:102063. 10.1016/j.isci.2021.102063 10.1016/j.isci.2021.102063 PMC7856464 33554067

[CR95] Ko A, Kang G, Hattler JB, Galadima HI, Zhang J, Li Q, Kim WK (2019). Macrophages but not astrocytes harbor HIV DNA in the brains of HIV-1-infected aviremic individuals on suppressive antiretroviral therapy. J Neuroimmune Pharmacol.

[CR96] Konishi H, Kobayashi M, Kunisawa T, Imai K, Sayo A, Malissen B, Crocker PR, Sato K, Kiyama H (2017). Siglec-H is a microglia-specific marker that discriminates microglia from CNS-associated macrophages and CNS-infiltrating monocytes. Glia.

[CR97] Kracht L, Borggrewe M, Eskandar S, Brouwer N, de Sousa C, Lopes SM, Laman JD, Scherjon SA, Prins JR, Kooistra SM, Eggen BJL (2020). Human fetal microglia acquire homeostatic immune-sensing properties early in development. Science.

[CR98] Lamers SL, Rose R, Maidji E, Agsalda-Garcia M, Nolan DJ, Fogel GB, Salemi M, Garcia DL, Bracci P, Yong W, Commins D, Said J, Khanlou N, Hinkin CH, Sueiras MV, Mathisen G, Donovan S, Shiramizu B, Stoddart CA, McGrath MS, Singer EJ (2016). HIV DNA is frequently present within pathologic tissues evaluated at autopsy from combined antiretroviral therapy-treated patients with undetectable viral loads. J Virol.

[CR99] Lebbink RJ, De Jong DCM, Wolters F, Kruse EM, Van Ham PM, Wiertz EJHJ, Nijhuis M (2017). A combinational CRISPR/Cas9 gene-editing approach can halt HIV replication and prevent viral escape. Sci Rep.

[CR100] Lee SC, Hatch WC, Liu W, Brosnan CF, Dickson DW (1993). Productive infection of human fetal microglia in vitro by HIV-1. Ann N Y Acad Sci.

[CR101] Lee SC, Liu W, Brosnan CF, Dickson DW (1992). Characterization of primary human fetal dissociated central nervous system cultures with an emphasis on microglia. Lab Investig.

[CR102] Lee YB, Nagai A, Kim SU (2002). Cytokines, chemokines, and cytokine receptors in human microglia. J Neurosci Res.

[CR103] Leek JT, Johnson WE, Parker HS, Jaffe AE, Storey JD (2012). The SVA package for removing batch effects and other unwanted variation in high-throughput experiments. Bioinformatics.

[CR104] Leone C, Le Pavec G, Même W, Porcheray F, Samah B, Dormont D, Gras G (2006). Characterization of human monocyte-derived microglia-like cells. Glia.

[CR105] Levtova N, Healy LM, Gonczi CMC, Stopnicki B, Blain M, Kennedy TE, Moore CS, Antel JP, Darlington PJ (2017). Comparative morphology and phagocytic capacity of primary human adult microglia with time-lapse imaging. J Neuroimmunol.

[CR106] Li Q, Barres BA (2018). Microglia and macrophages in brain homeostasis and disease. Nat Rev Immunol.

[CR107] Lin YT, Seo J, Gao F, Feldman HM, Wen HL, Penney J, Cam HP, Gjoneska E, Raja WK, Cheng J, Rueda R, Kritskiy O, Abdurrob F, Peng Z, Milo B, Yu CJ, Elmsaouri S, Dey D, Ko T, Yankner BA, Tsai LH (2018). APOE4 causes widespread molecular and cellular alterations associated with Alzheimer’s disease phenotypes in human iPSC-derived brain cell types. Neuron.

[CR108] Lisi L, Laudati E, Miscioscia TF, Dello Russo C, Topai A, Navarra P (2016). Antiretrovirals inhibit arginase in human microglia. J Neurochem.

[CR109] Lopes K de P, Snijders GJL, Humphrey J, Allan A, Sneeboer M, Navarro E, Schilder BM, Vialle RA, Parks M, Missall R, Zuiden W van, Gigase F, Kübler R, Berlekom AB van, Böttcher C, Priller J, Kahn RS, Witte LD de, Raj T (2020) Atlas of genetic effects in human microglia transcriptome across brain regions, aging and disease pathologies. BioRxiv 2020.10.27.356113. 10.1101/2020.10.27.356113

[CR110] Lue LF, Beach TG, Walker DG (2019). Alzheimer’s disease research using human microglia. Cells.

[CR111] Malikov V, Naghavi MH (2017). Localized phosphorylation of a kinesin-1 adaptor by a capsid-associated kinase regulates HIV-1 motility and uncoating. Cell Rep.

[CR112] Mamik MK, Ghorpade A (2014). Chemokine CXCL8 promotes HIV-1 replication in human monocyte-derived macrophages and primary microglia via nuclear factor-κB pathway. PLoS One.

[CR113] Mancuso R, Van Den Daele J, Fattorelli N, Wolfs L, Balusu S, Burton O, Liston A, Sierksma A, Fourne Y, Poovathingal S, Arranz-Mendiguren A, Sala Frigerio C, Claes C, Serneels L, Theys T, Perry VH, Verfaillie C, Fiers M, De Strooper B (2019). Stem-cell-derived human microglia transplanted in mouse brain to study human disease. Nat Neurosci.

[CR114] Marsh SE, Kamath T, Walker AJ, Dissing-Olesen L, Hammond TR, Young AMH, Abdulraouf A, Nadaf N, Dufort C, Murphy S, Kozareva V, Vanderburg C, Hong S, Bulstrode H, Hutchinson PJ, Gaffney DJ, Franklin RJM, Macosko EZ, Stevens B (2020). Single cell sequencing reveals glial specific responses to tissue processing & enzymatic dissociation in mice and humans. Available from bioRxiv.

[CR115] McQuade A, Coburn M, Tu CH, Hasselmann J, Davtyan H, Blurton-Jones M (2018). Development and validation of a simplified method to generate human microglia from pluripotent stem cells. Mol Neurodegener.

[CR116] Melief J, Koning N, Schuurman KG, Van De Garde MDB, Smolders J, Hoek RM, Van Eijk M, Hamann J, Huitinga I (2012). Phenotyping primary human microglia: tight regulation of LPS responsiveness. Glia.

[CR117] Melief J, Sneeboer MAM, Litjens M, Ormel PR, Palmen SJMC, Huitinga I, Kahn RS, Hol EM, de Witte LD (2016). Characterizing primary human microglia: a comparative study with myeloid subsets and culture models. Glia.

[CR118] Mildner A, Huang H, Radke J, Stenzel W, Priller J (2017). P2Y12 receptor is expressed on human microglia under physiological conditions throughout development and is sensitive to neuroinflammatory diseases. Glia.

[CR119] Mishra R, Chhatbar C, Singh S (2012). HIV-1 Tat C-mediated regulation of tumor necrosis factor receptor-associated factor-3 by microRNA 32 in human microglia. J Neuroinflammation.

[CR120] Mizee MR, Miedema SSM, van der Poel M, Adelia SKG, van Strien ME, Melief J, Smolders J, Hendrickx DA, Heutinck KM, Hamann J, Huitinga I (2017). Isolation of primary microglia from the human post-mortem brain: effects of ante- and post-mortem variables. Acta Neuropathol Commun.

[CR121] Muffat J, Li Y, Yuan B, Mitalipova M, Omer A, Corcoran S, Bakiasi G, Tsai LH, Aubourg P, Ransohoff RM, Jaenisch R (2016). Efficient derivation of microglia-like cells from human pluripotent stem cells. Nat Med.

[CR122] Nagai A, Nakagawa E, Hatori K, Choi HB, McLarnon JG, Lee MA, Kim SU (2001). Generation and characterization of immortalized human microglial cell lines: expression of cytokines and chemokines. Neurobiol Dis.

[CR123] Nandi S, Gokhan S, Dai XM, Wei S, Enikolopov G, Lin H, Mehler MF, Richard Stanley E (2012). The CSF-1 receptor ligands IL-34 and CSF-1 exhibit distinct developmental brain expression patterns and regulate neural progenitor cell maintenance and maturation. Dev Biol.

[CR124] Nebuloni M, Pellegrinelli A, Ferri A, Tosoni A, Bonetto S, Zerbi P, Boldorini R, Vago L, Costanzi G (2000). Etiology of microglial nodules in brains of patients with acquired immunodeficiency syndrome. J Neurovirol.

[CR125] Neumann H, Takahashi K (2007). Essential role of the microglial triggering receptor expressed on myeloid cells-2 (TREM2) for central nervous tissue immune homeostasis. J Neuroimmunol.

[CR126] Nimmerjahn A, Kirchhoff F, Helmchen F (2005). Neuroscience: resting microglial cells are highly dynamic surveillants of brain parenchyma in vivo. Science.

[CR127] Nosik M, Lavrov V, Svitich O (2021). HIV infection and related mental disorders. Brain Sci.

[CR128] Noto D, Sakuma H, Takahashi K, Saika R, Saga R, Yamada M, Yamamura T, Miyake S (2014). Development of a culture system to induce microglia-like cells from haematopoietic cells. Neuropathol Appl Neurobiol.

[CR129] Ohgidani M, Kato TA, Setoyama D, Sagata N, Hashimoto R, Shigenobu K, Yoshida T, Hayakawa K, Shimokawa N, Miura D, Utsumi H, Kanba S (2014). Direct induction of ramified microglia-like cells from human monocytes: dynamic microglial dysfunction in Nasu-Hakola disease. Sci Rep.

[CR130] Olah M, Raj D, Brouwer N, De Haas AH, Eggen BJL, Den Dunnen WFA, Biber KPH, Boddeke HWGM (2012). An optimized protocol for the acute isolation of human microglia from autopsy brain samples. Glia.

[CR131] Ormel PR, Böttcher C, Gigase FAJ, Missall RD, van Zuiden W, Fernández Zapata MC, Ilhan D, de Goeij M, Udine E, Sommer IEC, Priller J, Raj T, Kahn RS, Hol EM, de Witte LD (2020). A characterization of the molecular phenotype and inflammatory response of schizophrenia patient-derived microglia-like cells. Brain Behav Immun.

[CR132] Ormel PR, Vieira de Sá R, van Bodegraven EJ, Karst H, Harschnitz O, Sneeboer MAM, Johansen LE, van Dijk RE, Scheefhals N, Berdenis van Berlekom A, Ribes Martínez E, Kling S, MacGillavry HD, van den Berg LH, Kahn RS, Hol EM, de Witte LD, Pasterkamp RJ (2018). Microglia innately develop within cerebral organoids. Nat Commun.

[CR133] Pandya H, Shen MJ, Ichikawa DM, Sedlock AB, Choi Y, Johnson KR, Kim G, Brown MA, Elkahloun AG, Maric D, Sweeney CL, Gossa S, Malech HL, McGavern DB, Park JK (2017). Differentiation of human and murine induced pluripotent stem cells to microglia-like cells. Nat Neurosci.

[CR134] Park J, Wetzel I, Marriott I, Dréau D, D’Avanzo C, Kim DY, Tanzi RE, Cho H (2018). A 3D human triculture system modeling neurodegeneration and neuroinflammation in Alzheimer’s disease. Nat Neurosci.

[CR135] Patel AB, Tsilioni I, Leeman SE, Theoharides TC (2016). Neurotensin stimulates sortilin and mTOR in human microglia inhibitable by methoxyluteolin, a potential therapeutic target for autism. Proc Natl Acad Sci U S A.

[CR136] Patir A, Shih B, McColl BW, Freeman TC (2019). A core transcriptional signature of human microglia: derivation and utility in describing region-dependent alterations associated with Alzheimer’s disease. Glia.

[CR137] Pilcher CD, Shugars DC, Fiscus SA, Miller WC, Menezes P, Giner J, Dean B, Robertson K, Hart CE, Lennox JL, Eron JJ, Hicks CB (2001). HIV in body fluids during primary HIV infection: implications for pathogenesis, treatment and public health. AIDS.

[CR138] Plemel JR, Stratton JA, Michaels NJ, Rawji KS, Zhang E, Sinha S, Baaklini CS, Dong Y, Ho M, Thorburn K, Friedman TN, Jawad S, Silva C, Caprariello AV, Hoghooghi V, Yue J, Jaffer A, Lee K, Kerr BJ, Midha R, Stys PK, Biernaskie J, Yong VW (2020) Microglia response following acute demyelination is heterogeneous and limits infiltrating macrophage dispersion. Sci Adv 6:eaay6324. 10.1126/sciadv.aay6324 10.1126/sciadv.aay6324 PMC6962036 31998844

[CR139] Qian X, Song H, Ming G-L (2019) Brain organoids: advances, applications and challenges. Development 146: dev166074. 10.1242/dev.166074 10.1242/dev.166074 PMC6503989 30992274

[CR140] Rai MA, Hammonds J, Pujato M, Mayhew C, Roskin K, Spearman P (2020). Comparative analysis of human microglial models for studies of HIV replication and pathogenesis. Retrovirology.

[CR141] Rawat P, Spector SA (2017). Development and characterization of a human microglia cell model of HIV-1 infection. J Neurovirol.

[CR142] Rawat P, Teodorof-Diedrich C, Spector SA (2019). Human immunodeficiency virus type-1 single-stranded RNA activates the NLRP3 inflammasome and impairs autophagic clearance of damaged mitochondria in human microglia. Glia.

[CR143] Ritzel RM, Patel AR, Grenier JM, Crapser J, Verma R, Jellison ER, McCullough LD (2015). Functional differences between microglia and monocytes after ischemic stroke. J Neuroinflammation.

[CR144] Robinson MD, McCarthy DJ, Smyth GK (2009). edgeR: a Bioconductor package for differential expression analysis of digital gene expression data. Bioinformatics.

[CR145] Rodríguez-Gómez JA, Kavanagh E, Engskog-Vlachos P, Engskog MKR, Herrera AJ, Espinosa-Oliva AM, Joseph B, Hajji N, Venero JL, Burguillos MA (2020). Microglia: agents of the CNS pro-inflammatory response. Cells.

[CR146] Rustenhoven J, Park TI-H, Schweder P, Scotter J, Correia J, Smith AM, Gibbons HM, Oldfield RL, Bergin PS, Mee EW, Faull RLM, Curtis MA, Scott Graham E, Dragunow M (2016). Isolation of highly enriched primary human microglia for functional studies. Sci Rep.

[CR147] Ryan KJ, White CC, Patel K, Xu J, Olah M, Replogle JM, Frangieh M, Cimpean M, Winn P, McHenry A, Kaskow BJ, Chan G, Cuerdon N, Bennett DA, Boyd JD, Imitola J, Elyaman W, De Jager PL, Bradshaw EM (2017) A human microglia-like cellular model for assessing the effects of neurodegenerative disease gene variants. Sci Transl Med 9: eaai7635. 10.1126/scitranslmed.aai7635 10.1126/scitranslmed.aai7635 PMC5945290 29263232

[CR148] Ryan SK, Gonzalez MV, Garifallou JP, Bennett FC, Williams KS, Sotuyo NP, Mironets E, Cook K, Hakonarson H, Anderson SA, Jordan-Sciutto KL (2020). Neuroinflammation and EIF2 signaling persist despite antiretroviral treatment in an hiPSC tri-culture model of HIV infection. Stem Cell Reports.

[CR149] Salamanca L, Mechawar N, Murai KK, Balling R, Bouvier DS, Skupin A (2019). MIC-MAC: an automated pipeline for high-throughput characterization and classification of three-dimensional microglia morphologies in mouse and human postmortem brain samples. Glia.

[CR150] Samikkannu T, Atluri VSR, Nair MPN (2016). HIV and cocaine impact glial metabolism: energy sensor AMP-activated protein kinase role in mitochondrial biogenesis and epigenetic remodeling. Sci Rep.

[CR151] Sasaki Y, Ohsawa K, Kanazawa H, Kohsaka S, Imai Y (2001). Iba1 is an actin-cross-linking protein in macrophages/microglia. Biochem Biophys Res Commun.

[CR152] Satoh JI, Kino Y, Asahina N, Takitani M, Miyoshi J, Ishida T, Saito Y (2016). TMEM119 marks a subset of microglia in the human brain. Neuropathology.

[CR153] Schuenke K, Gelman BB (2003). Human microglial cell isolation from adult autopsy brain: brain pH, regional variation, and infection with human immunodeficiency virus type 1. J Neurovirol.

[CR154] Sellgren CM, Gracias J, Watmuff B, Biag JD, Thanos JM, Whittredge PB, Fu T, Worringer K, Brown HE, Wang J, Kaykas A, Karmacharya R, Goold CP, Sheridan SD, Perlis RH (2019). Increased synapse elimination by microglia in schizophrenia patient-derived models of synaptic pruning. Nat Neurosci.

[CR155] Sellgren CM, Sheridan SD, Gracias J, Xuan D, Fu T, Perlis RH (2017). Patient-specific models of microglia-mediated engulfment of synapses and neural progenitors. Mol Psychiatry.

[CR156] Sengupta S, Siliciano RF (2018). Targeting the latent reservoir for HIV-1. Immunity.

[CR157] Sidhaye J, Knoblich JA (2021). Brain organoids: an ensemble of bioassays to investigate human neurodevelopment and disease. Cell Death Differ.

[CR158] Smyth GK (2005) limma: Linear Models for Microarray Data. In: Gentleman R., Carey V.J., Huber W., Irizarry R.A., Dudoit S. (eds) Bioinformatics and computational biology solutions using R and Bioconductor. Statistics for Biology and Health. Springer, New York, NY. 10.1007/0-387-29362-0_23

[CR159] Sneeboer MAM, Snijders GJLJ, Berdowski WM, Fernández-Andreu A, van Mierlo HC, Berdenis van Berlekom A, Litjens M, Kahn RS, Hol EM, de Witte LD (2019). Microglia in post-mortem brain tissue of patients with bipolar disorder are not immune activated. Transl Psychiatry.

[CR160] Speicher AM, Wiendl H, Meuth SG, Pawlowski M (2019). Generating microglia from human pluripotent stem cells: novel in vitro models for the study of neurodegeneration. Mol Neurodegener.

[CR161] Stegle O, Teichmann SA, Marioni JC (2015). Computational and analytical challenges in single-cell transcriptomics. Nat Rev Genet.

[CR162] Stence N, Waite M, Dailey ME (2001). Dynamics of microglial activation: a confocal time-lapse analysis in hippocampal slices. Glia.

[CR163] Strizki JM, Albright AV, Sheng H, O’Connor M, Perrin L, González-Scarano F (1996). Infection of primary human microglia and monocyte-derived macrophages with human immunodeficiency virus type 1 isolates: evidence of differential tropism. J Virol.

[CR164] Suzuki S, Tobiume M, Kameoka M, Sato K, Takahashi TA, Mukai T, Ikuta K (2000). Exposure of normal monocyte-derived dendritic cells to human immunodeficiency virus type-1 particles leads to the induction of apoptosis in co-cultured CD4+ as well as CD8+ T cells. Microbiol Immunol.

[CR165] Svoboda DS, Barrasa MI, Shu J, Rietjens R, Zhang S, Mitalipova M, Berube P, Fu D, Shultz LD, Bell GW, Jaenisch R (2019). Human iPSC-derived microglia assume a primary microglia-like state after transplantation into the neonatal mouse brain. Proc Natl Acad Sci U S A.

[CR166] Takata K, Kozaki T, Lee CZW, Thion MS, Otsuka M, Lim S, Utami KH, Fidan K, Park DS, Malleret B, Chakarov S, See P, Low D, Low G, Garcia-Miralles M, Zeng R, Zhang J, Goh CC, Gul A, Hubert S, Lee B, Chen J, Low I, Shadan NB, Lum J, Wei TS, Mok E, Kawanishi S, Kitamura Y, Larbi A, Poidinger M, Renia L, Ng LG, Wolf Y, Jung S, Önder T, Newell E, Huber T, Ashihara E, Garel S, Pouladi MA, Ginhoux F (2017). Induced-pluripotent-stem-cell-derived primitive macrophages provide a platform for modeling tissue-resident macrophage differentiation and function. Immunity.

[CR167] Tambussi G, Gori A, Capiluppi B, Balotta C, Papagno L, Morandini B, Di Pietro M, Ciuffreda D, Saracco A, Lazzarin A (2000). Neurological symptoms during primary human immunodeficiency virus (HIV) infection correlate with high levels of HIV RNA in cerebrospinal fluid. Clin Infect Dis.

[CR168] Tatro ET, Soontornniyomkij B, Letendre SL, Achim CL (2014). Cytokine secretion from brain macrophages infected with human immunodeficiency virus in vitro and treated with raltegravir. BMC Infect Dis.

[CR169] Taya K, Nakayama EE, Shioda T (2014). Moderate restriction of macrophage-tropic human immunodeficiency virus type 1 by SAMHD1 in monocyte-derived macrophages. PLoS One.

[CR170] Tewari M, Khan M, Verma M, Coppens J, Kemp JM, Bucholz R, Mercier P, Egan TM (2021). Physiology of cultured human microglia maintained in a defined culture medium. ImmunoHorizons.

[CR171] Thompson KA, Cherry CL, Bell JE, McLean CA (2011). Brain cell reservoirs of latent virus in presymptomatic HIV-infected individuals. Am J Pathol.

[CR172] Timmerman R, Burm SM, Bajramovic JJ (2018). An overview of in vitro methods to study microglia. Front Cell Neurosci.

[CR173] Tomitaka A, Arami H, Huang Z, Raymond A, Rodriguez E, Cai Y, Febo M, Takemura Y, Nair M (2018). Hybrid magneto-plasmonic liposomes for multimodal image-guided and brain-targeted HIV treatment. Nanoscale.

[CR174] Torres-Platas SG, Comeau S, Rachalski A, Bo GD, Cruceanu C, Turecki G, Giros B, Mechawar N (2014). Morphometric characterization of microglial phenotypes in human cerebral cortex. J Neuroinflammation.

[CR175] Trillo-Pazos G, Diamanturos A, Rislove L, Menza T, Chao W, Belem P, Sadiq S, Morgello S, Sharer L, Volsky DJ (2003). Detection of HIV-1 DNA in microglia/macrophages, astrocytes and neurons isolated from brain tissue with HIV-1 encephalitis by laser capture microdissection. Brain Pathol.

[CR176] Tso FY, Kang G, Kwon EH, Julius P, Li Q, West JT, Wood C (2018). Brain is a potential sanctuary for subtype C HIV-1 irrespective of ART treatment outcome. PLoS One.

[CR177] Tsunetsugu-Yokota Y, Akagawa K, Kimoto H, Suzuki K, Iwasaki M, Yasuda S, Häusser G, Hultgren C, Meyerhans A, Takemori T (1995). Monocyte-derived cultured dendritic cells are susceptible to human immunodeficiency virus infection and transmit virus to resting T cells in the process of nominal antigen presentation. J Virol.

[CR178] Tuttle DL, Harrison JK, Anders C, Sleasman JW, Goodenow MM (1998). Expression of CCR5 increases during monocyte differentiation and directly mediates macrophage susceptibility to infection by human immunodeficiency virus type 1. J Virol.

[CR179] Ulfhammer G, Eden A, Mellgren S, Fuchs D, Zetterberg H, Hagberg L, Nilsson S, Yilmaz A, Gisslen M (2018). Persistent central nervous system immune activation following more than 10 years of effective HIV antiretroviral treatment. AIDS.

[CR180] Van Maarseveen NM, Huigen MCDG, De Jong D, Smits AM, Boucher CAB, Nijhuis M (2006). A novel real-time PCR assay to determine relative replication capacity for HIV-1 protease variants and/or reverse transcriptase variants. J Virol Methods.

[CR181] Walker FR, Beynon SB, Jones KA, Zhao Z, Kongsui R, Cairns M, Nilsson M (2014). Dynamic structural remodelling of microglia in health and disease: a review of the models, the signals and the mechanisms. Brain Behav Immun.

[CR182] Wallet C, De Rovere M, Van Assche J, Daouad F, De Wit S, Gautier V, Mallon PWG, Marcello A, Van Lint C, Rohr O, Schwartz C (2019). Microglial cells: the main HIV-1 reservoir in the brain. Front Cell Infect Microbiol.

[CR183] Wang Y, Liu M, Lu Q, Farrell M, Lappin JM, Shi J, Lu L, Bao Y (2020). Global prevalence and burden of HIV-associated neurocognitive disorder: a meta-analysis. Neurology.

[CR184] Watkins BA, Dorn HH, Kelly WB, Armstrong RC, Potts BJ, Michaels F, Kufta CV, Dubois-Dalcq M (1990). Specific tropism of HIV-1 for microglial cells in primary human brain cultures. Science.

[CR185] Winston A, Spudich S (2020). Cognitive disorders in people living with HIV. Lancet HIV.

[CR186] Wires ES, Alvarez D, Dobrowolski C, Wang Y, Morales M, Karn J, Harvey BK (2012). Methamphetamine activates nuclear factor kappa-light-chain-enhancer of activated B cells (NF-κB) and induces human immunodeficiency virus (HIV) transcription in human microglial cells. J Neurovirol.

[CR187] Wolf SA, Boddeke HWGM, Kettenmann H (2017). Microglia in physiology and disease. Annu Rev Physiol.

[CR188] Xu M, Zhang L, Liu G, Jiang N, Zhou W, Zhang Y (2019). Pathological changes in Alzheimer’s disease analyzed using induced pluripotent stem cell-derived human microglia-like cells. J Alzheimer’s Dis.

[CR189] Yamasaki R, Lu H, Butovsky O, Ohno N, Rietsch AM, Cialic R, Wu PM, Doykan CE, Lin J, Cotleur AC, Kidd G, Zorlu MM, Sun N, Hu W, Liu LP, Lee JC, Taylor SE, Uehlein L, Dixon D, Gu J, Floruta CM, Zhu M, Charo IF, Weiner HL, Ransohoff RM (2014). Differential roles of microglia and monocytes in the inflamed central nervous system. J Exp Med.

[CR190] Zenón F, Cantres-Rosario Y, Adiga R, Gonzalez M, Rodriguez-Franco E, Langford D, Melendez LM (2015). HIV-infected microglia mediate cathepsin B-induced neurotoxicity. J Neurovirol.

[CR191] Zhang Y, Sloan SA, Clarke LE, Caneda C, Plaza CA, Blumenthal PD, Vogel H, Steinberg GK, Edwards MSB, Li G, Duncan JA, Cheshier SH, Shuer LM, Chang EF, Grant GA, Gephart MGH, Barres BA (2016). Purification and characterization of progenitor and mature human astrocytes reveals transcriptional and functional differences with mouse. Neuron.

[CR192] Zhou LJ, Tedder TF (1996). CD14+ blood monocytes can differentiate into functionally mature CD83+ dendritic cells. Proc Natl Acad Sci U S A.

