# Supplementary material for: Human microglial models to study HIV infection and neuropathogenesis: a literature overview and comparative analyses
Source: J Neurovirol. 2022 Feb 9;28(1):64–91. doi: 10.1007/s13365-021-01049-w (PMC9076745; doi:10.1007/s13365-021-01049-w)
Supplement: Supplementary file 1 — Supplementary file1 (PDF 591 kb) [file 13365_2021_1049_MOESM1_ESM.pdf]

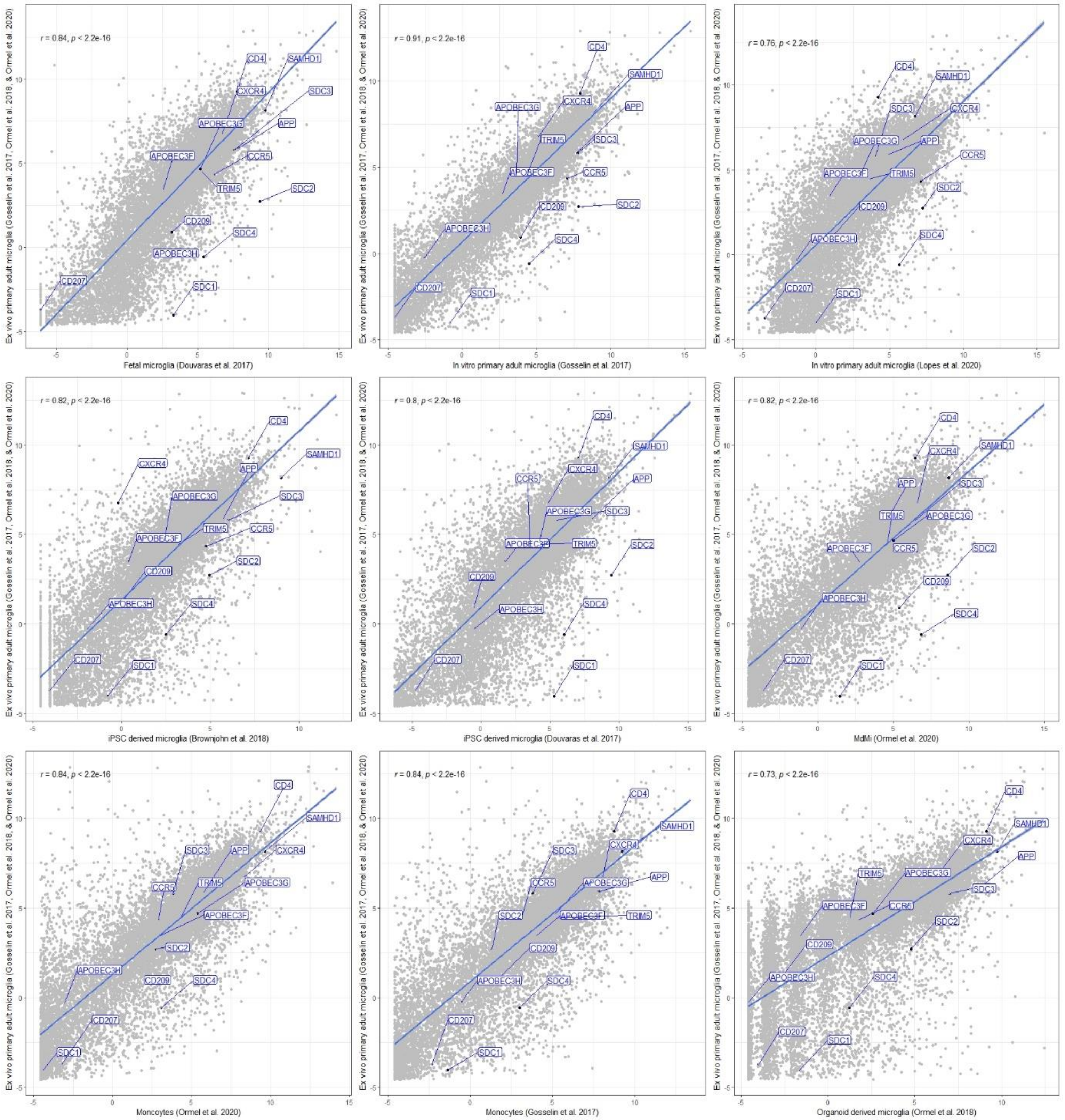

Figure S1: Regression coefficients of the full transcriptome datasets of primary microglia and the different microglia culture models.

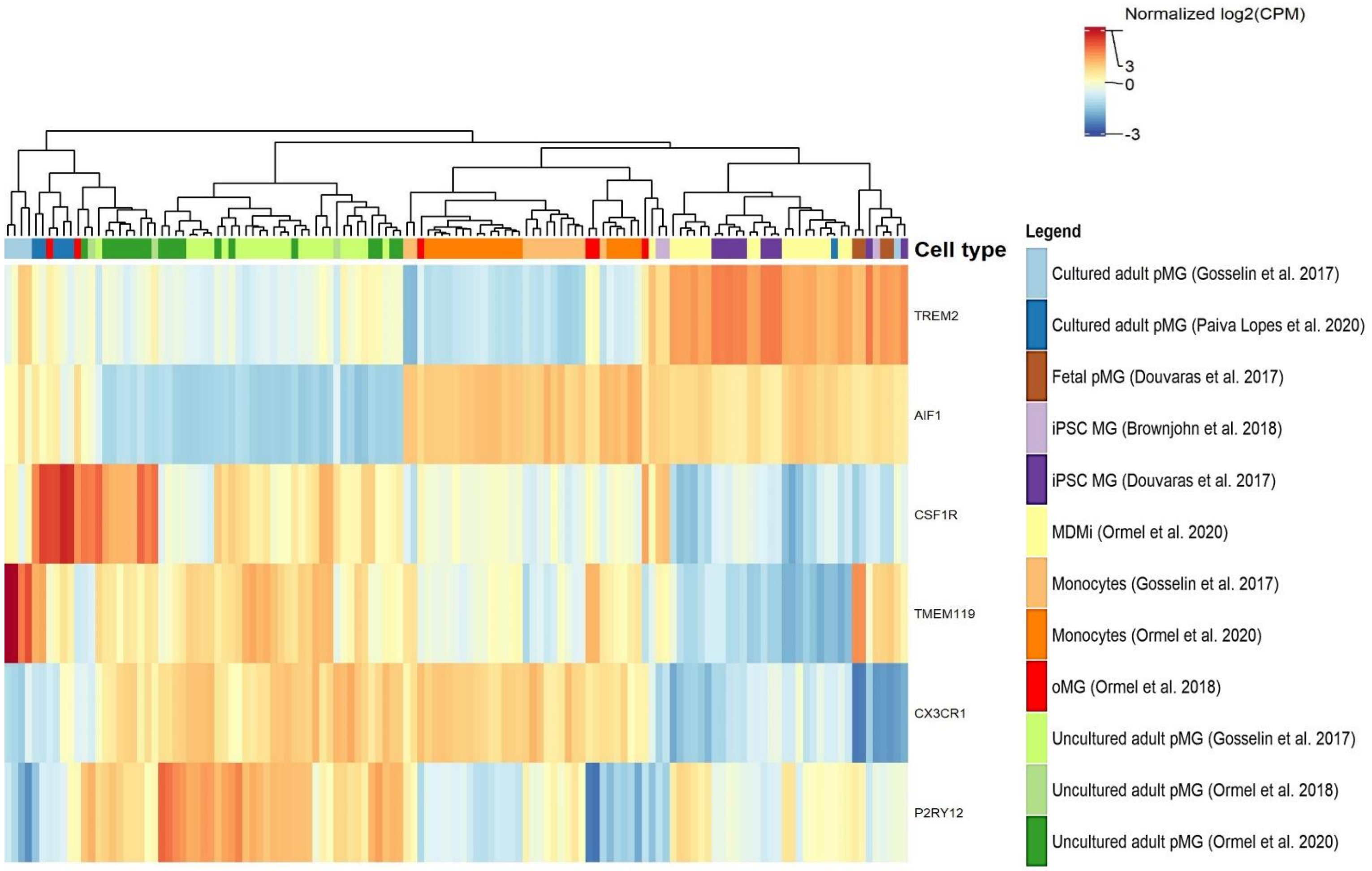

Figure S2: Gene expression analysis of microglia culture models on microglia-specific genes. Heatmap of Pearson r for between each cell type (cluster distances are Euclidean).

Supplementary Table 1: Primer sequences used for qRT-PCR experiments

Gene	5' forward 3'	5' reverse 3'
**CD4**	TCCAGAGGCTTAATCACACCG	GGCTAGGCTTGAAGGAAAAGG
**CXCR4**	ACTGTTGTCTGAACCCCATCC	AGAGGTGAGTGCGTGCTGG
**CCR5**	TTATACATCGGAGCCCTGCC	ATCAGGATGAGGATGACCAGC
**GAPDH**	TGTTCGACAGTCAGCCGCATCTTC	CAGAGTTAAAAGCAGCCCTGGTGA
